# A Novel Cultivation System for Germ Cell Proliferation and Sustaining Whole Testicular Niche

**DOI:** 10.1002/advs.202502435

**Published:** 2025-07-28

**Authors:** Yu Xia, Xiaoxuan Zhang, Bohang Zhang, Minhui Lu, Qing Cheng, Li Liu, Lifang Shi, Yiqiang Cui, Dingdong Chen, Laihua Li, Xuejiang Guo, Jingtao Guo, Jiahao Sha, Yuanjin Zhao, Yan Yuan

**Affiliations:** ^1^ State Key Laboratory of Reproductive Medicine and Offspring Health Nanjing Medical University Nanjing Jiangsu 211166 China; ^2^ Department of Andrology, Nanjing Drum Tower Hospital, School of Biological Science and Medical Engineering Southeast University Nanjing 210096 China; ^3^ Women's Hospital of Nanjing Medical University Nanjing Women and Children's Healthcare Hospital Nanjing 210000 China; ^4^ The Affiliated Taizhou People's Hospital of Nanjing Medical University, Taizhou School of Clinical Medicine Nanjing Medical University Nanjing Jiangsu 211166 China; ^5^ State Key Laboratory of Stem Cell and Reproductive Biology, Institute of Zoology Chinese Academy of Sciences Beijing 100101 China; ^6^ State Key Laboratory of Reproductive Medicine and Offspring Health, Women's Hospital of Nanjing Medical University, Nanjing Maternity and Child Health Care Hospital Nanjing Medical University Nanjing Jiangsu 211166 China

**Keywords:** fertility preservation, in vitro organ culture, in vitro spermatogenesis, microneedle, spermatogonial proliferation

## Abstract

Childhood cancer treatments often impair male fertility due to gonadotoxic effects. While in vitro culture of prepubertal testis tissue offers a potential solution to preserve fertility, producing sufficient sperm remains a major barrier. To address this, we developed a hydrogel microneedle‐based culture system, which is used to culture mouse testes from 5 days postpartum (d*pp*) and establish a model of ‘whole testicular spermatogonia pool’ (WTSP). This system promotes over fourfold expansion of undifferentiated spermatogonia, compared to declining numbers during in vivo development. Transplantation of WTSP into nude mice doubled spermatids count per tubule compared to conventional whole testes transplantation. Furthermore, in vitro meiosis induction of WTSP significantly enhanced spermatid proportion, thus generating fertile offspring. Using this system, we also cultured the gonads harvested from aborted human male fetuses and observes significant proliferation of spermatogonia. Lastly, it is shown that the cellular states of the WTSP closely resemble those of 5 d*pp* mouse testes, and the role of EPHA2 in promoting spermatogonia proliferation by activating the PI3K‐AKT‐mTOR pathway. In conclusion, the WTSP offers a promising method for preserving fertility in prepubertal male cancer patients by maintaining and expanding spermatogonia extracted before treatment.

## Graphical abstract

A hydrogel microneedle‐based system enables whole testicular cultivation, supporting spermatogonial proliferation and enhancing spermatogenesis in vitro. This ‘whole testicular spermatogonia pool’ (WTSP) significantly increases spermatid production and fertility potential in mice. This system also promotes spermatogonial expansion in human fetal testes, offering a promising strategy for fertility preservation in prepubertal male cancer patients.

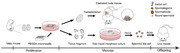

## 1 Introduction

Pediatric cancer is diagnosed in ≈1 in 400 children before age 15, with a higher incidence observed in male patients.^[^ ^1^ ^]^ Advances in cancer treatment have increased the 5‐year survival rate for these young patients to 85%.^[^ ^2^ ^]^ However, the radiation and chemotherapy treatments, often result in collateral damage to the male reproductive system. Approximately 30% of male patients undergoing these treatments experience azoospermia, while 18% face oligospermia and fertility issues.^[^ ^3^, ^4^ ^]^ Preserving fertility through sperm cryopreservation before treatment is the optimal choice for adult male cancer patients. However, this option is not available for prepubertal boys, as they have not yet undergone spermatogenesis. Therefore, preserving spermatogonia present in young male and reconstituting spermatogenesis constitutes a promising approach for these patients.^[^ ^5^, ^6^, ^7^ ^]^

Successful sperm retrieval through in vivo transplantation of testis tissue has been documented in mice and monkeys.^[^ ^8^, ^9^ ^]^ This method, while promising for addressing fertility preservation in prepubertal males, raises concerns when a potential risk exists of re‐introducing malignant cells.^[^ ^10^ ^]^ The development of in vitro platforms for generating spermatids offers an alternative for fertility preservation,^[^ ^11^, ^12^ ^]^ avoiding malignant cell contamination in the process.^[^ ^10^, ^13^ ^]^ Since the establishment of the testicular organotypic culture system in 1959,^[^ ^14^ ^]^ various culture systems have been developed.^[^ ^11^, ^12^, ^15^, ^16^, ^17^, ^18^ ^]^ Notably, these systems have been reported to successfully produce functional haploid germ cells both in mice^[^ ^11^ ^]^ and humans^[^ ^12^ ^]^ under in vitro conditions. However, the tissue obtained from testicular biopsies in prepubertal boys is minimal and contains only a limited number of spermatogonia.^[^ ^10^ ^]^ Additionally, inadequate nutrient supply often leads to cell death in the central region of cultured tissue.^[^ ^11^ ^]^ Therefore, in vitro expansion of spermatogonia and the enhancement of nutrient supply efficiency in testicular culture systems are critical challenges that must be addressed to advance fertility preservation for prepubertal boys.

Here, to address the issue during cultivation, we pioneered the use of hydrogel microneedles in organ culture. The hydrogel microneedle is known for its minimal invasiveness and precise delivery of biologically active substances, presents a novel solution.^[^ ^19^, ^20^, ^21^ ^]^ Using a polyethylene glycol diacrylate (PEGDA)‐hydrogel‐based microneedle, we developed an advanced culture system for whole mouse testis cultivation, which not only supports the proliferation of undifferentiated spermatogonia but also effectively addresses the issue of central tissue necrosis in cultured testes. Our system effectively preserves testicular niche, enabling the establishment of a highly efficient in vitro spermatogonia expansion platform—the “whole testicular spermatogonia pool” (WTSP). More importantly, using this system, we cultured 24‐week human embryonic testicular gonadal specimens. At this developmental stage, the spermatogonia exhibit characteristics similar to those of certain spermatogonia found in children and adults.^[^ ^22^, ^23^ ^]^ Notably, we observed significant spermatogonial proliferation, providing new insights into potential strategies for fertility preservation in prepubertal boys.

## 2 Results

### 2.1 Cultivation of Whole Mouse Testes Using PEGDA Hydrogel Microneedles

**Figure 1:**
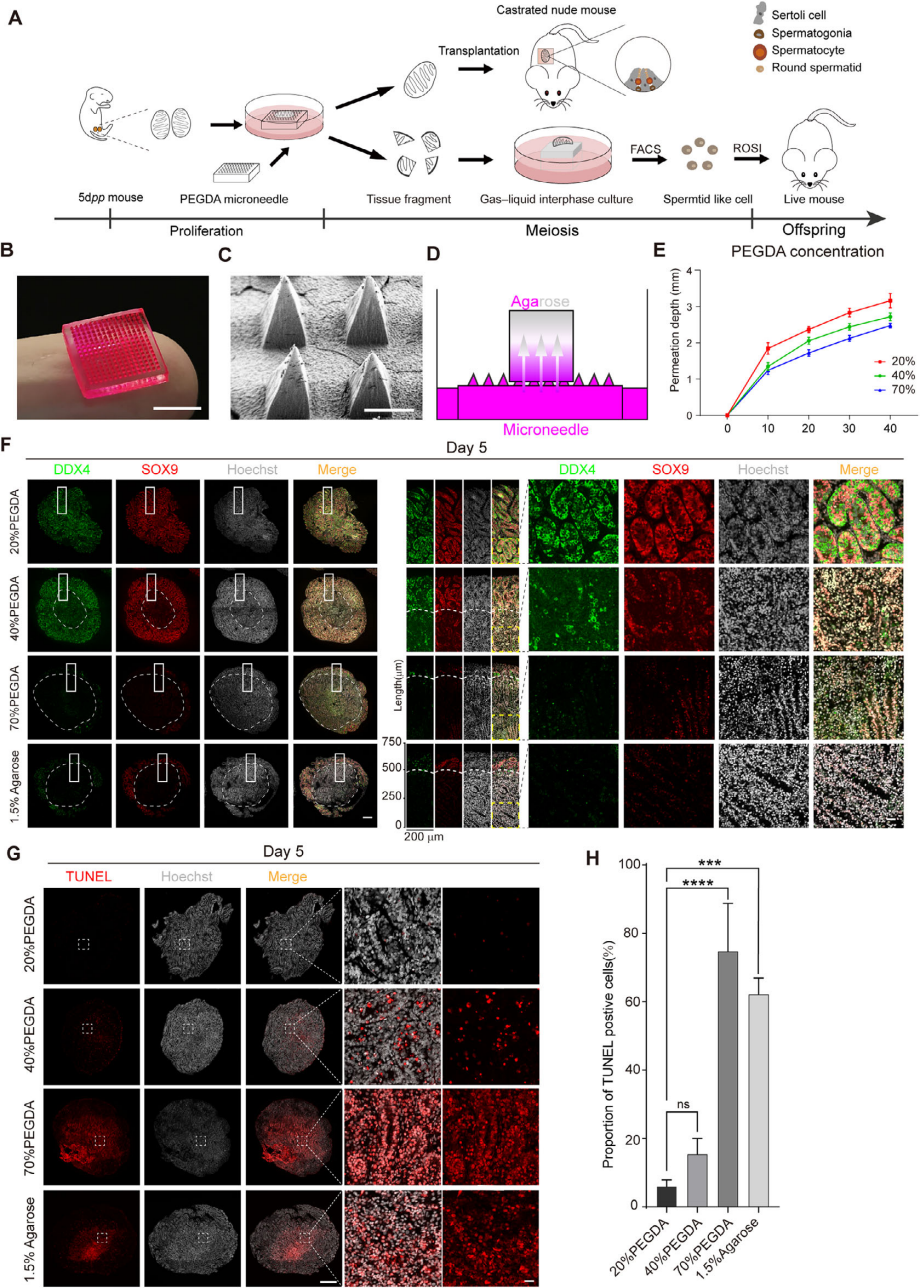
Cultivation of whole testes using PEGDA microneedles. A) Overview of study design and analyses. B) Optical image of PEGDA microneedles with rhodamine B. Scale bar, 5 mm. C) SEM image of PEGDA microneedles. Scale bar, 200 µm. D) Schematic representation of microneedles permeation mold. E) The permeation depth of rhodamine B in agarose by microneedles at different PEGDA concentrations. F) Immunofluorescence of the maximum cross‐section of testicular tissue with DDX4 (green), SOX9 (red). The nuclei were stained by Hoechst 33342 (grey). Scale bar, 200 µm. The insets show magnified views of testes. Scale bar of right panel, 20 µm. G) TUNEL staining (red) of the maximum cross‐section of testicular tissue with Hoechst 33342 stained nuclei (grey). Scale bar, left panel: 200 µm; right panel: 20 µm. H) The proportion of TUNEL‐positive cells to the total number of cells in a population. Data are shown as mean values ± s.d.; n = 3. ns, not significant. Statistical significance was assessed by one‐way ANOVA (40% PEGDA: *p* = 0.5157; 70% PEGDA: *p* < 0.0001; 1.5% Agarose: *p* = 0.0001).

To establish an efficient in vitro testis culture system, we designed microneedles by employing a conventional mold‐based strategy that combines PEGDA hydrogels (**Figure** **1**A). The PEGDA hydrogel microneedles mimic a vascular system by creating microchannels that enable direct nutrient and oxygen transport to the organ's interior.^[^ ^24^ ^]^ Additionally, the porous and loose structure of the hydrogel provides efficient pathways for material delivery, further supporting the cultured tissue.^[^ ^19^ ^]^ Morphological analysis using the colored reagent rhodamine B and scanning electron microscope (SEM), showed that the microneedles possess pyramid‐type tip (Figure 1B,C; Figure S1A, Supporting Information). SEM imaging also showed that the microneedles are characterized by a porous surface (Figure S1B, Supporting Information), a structural property that enhances substance release.^[^ ^25^, ^26^ ^]^

To evaluate the mechanical strength of PEGDA hydrogel microneedles, we performed stress tests at different concentrations. We found that higher PEGDA concentrations resulted in greater maximum stress capacity of the microneedles (Figure S1C, Supporting Information). Moreover, we found that microneedles maintained their sharp pyramid tip and overall integrity at a PEGDA concentration of 20% or higher (Figure S1D, Supporting Information). Importantly, we found that to successfully culture testis tissues, it was essential to use complete needle tips characterized by sufficient hardness. Based on these findings, for further experiments, we prepared microneedles at PEGDA concentrations of 20%, 40%, and 70%.

We next analyzed the permeability of rhodamine B applied through microneedles prepared at different PEGDA concentrations, assessing the performance of the microneedles as a release substrate (Figure 1D). We found that higher PEGDA concentrations reduced the permeation depth of rhodamine B in agarose, with microneedles of 20% PEGDA concentration showing the highest permeation speed, reaching nearly 3.2 millimeters in 40 min (Figure 1E; Figure S1E, Supporting Information). These results showed that microneedles produced at varying PEGDA concentrations possess capability for substance delivery, with 20% PEGDA microneedles having the highest permeation efficiency.

To validate the cultivation effects of different PEGDA concentrations, we subsequently utilized these 20%, 40%, and 70% PEGDA microneedles for culturing testicular tissue. In prepubertal children aged 8 years, germ cells are primarily spermatogonia and have not yet entered meiosis.^[^ ^27^ ^]^ Similarly, in 5 d*pp* mouse testes, the germ cells are predominantly undifferentiated and differentiating spermatogonia, without initiating meiotic division.^[^ ^28^ ^]^ Given this comparable germ cell state, we selected 5 d*pp* mouse testes as a model for 3D in vitro culture. After removing the tunica albuginea, the intact testes were gently placed on the tips of a PEGDA microneedle, which were partially immersed in the culture medium to support the cultivation process. We conducted cultures with varying concentrations of PEGDA (20%, 40%, and 70%) alongside control groups using 1.5% agarose gels (Figure S1F, Supporting Information).

Five days post cultivation (5 dpc), we assessed the viability of both germ cells (DDX4+) and Sertoli cells (SOX9+) in the cultured tissues using immunofluorescence. We found that cultivation using 20% PEGDA microneedles outperformed the traditional 1.5% agarose gel method, specifically in maintaining germ cell viability (Figure 1F; Figure S1G,H, Supporting Information). We also assessed the degree of apoptosis in testicular cells under different cultivation conditions at 5 dpc, we observed pronounced apoptosis in the central cells of tissues cultured with traditional 1.5% agarose and 70% PEGDA microneedles. Remarkably, the 20% PEGDA microneedle group displayed fewest TUNEL+ apoptotic cells, demonstrating it is more efficient in reducing apoptosis compared to other methods (Figure 1G,H; Figure S1I, Supporting Information). Lastly, to confirm that the 20% PEGDA microneedles do not affect cell growth, we co‐cultured them with NIH‐3T3 cells. We found that the growth of these cells was comparable to the growth of our control cells that were not co‐cultured with the 20% PEGDA microneedles. These findings suggested that the PEGDA microneedles are non‐toxic and biocompatible (Figure S1J,K, Supporting Information).

Together, these findings showed that using 20% PEGDA microneedles allows for effective medium permeation during testis cultivation. The capacity of 20% PEGDA microneedles to deliver substances at high efficiency significantly improves the nutrient supply to cultured testes. As a result, central necrosis typically seen in conventional in vitro testis cultures is prevented, thus allowing for the maintenance of the whole testis during culturing.

### 2.2 Establishment of the Whole Testicular Spermatogonia Pool (WTSP)

**Figure 2:**
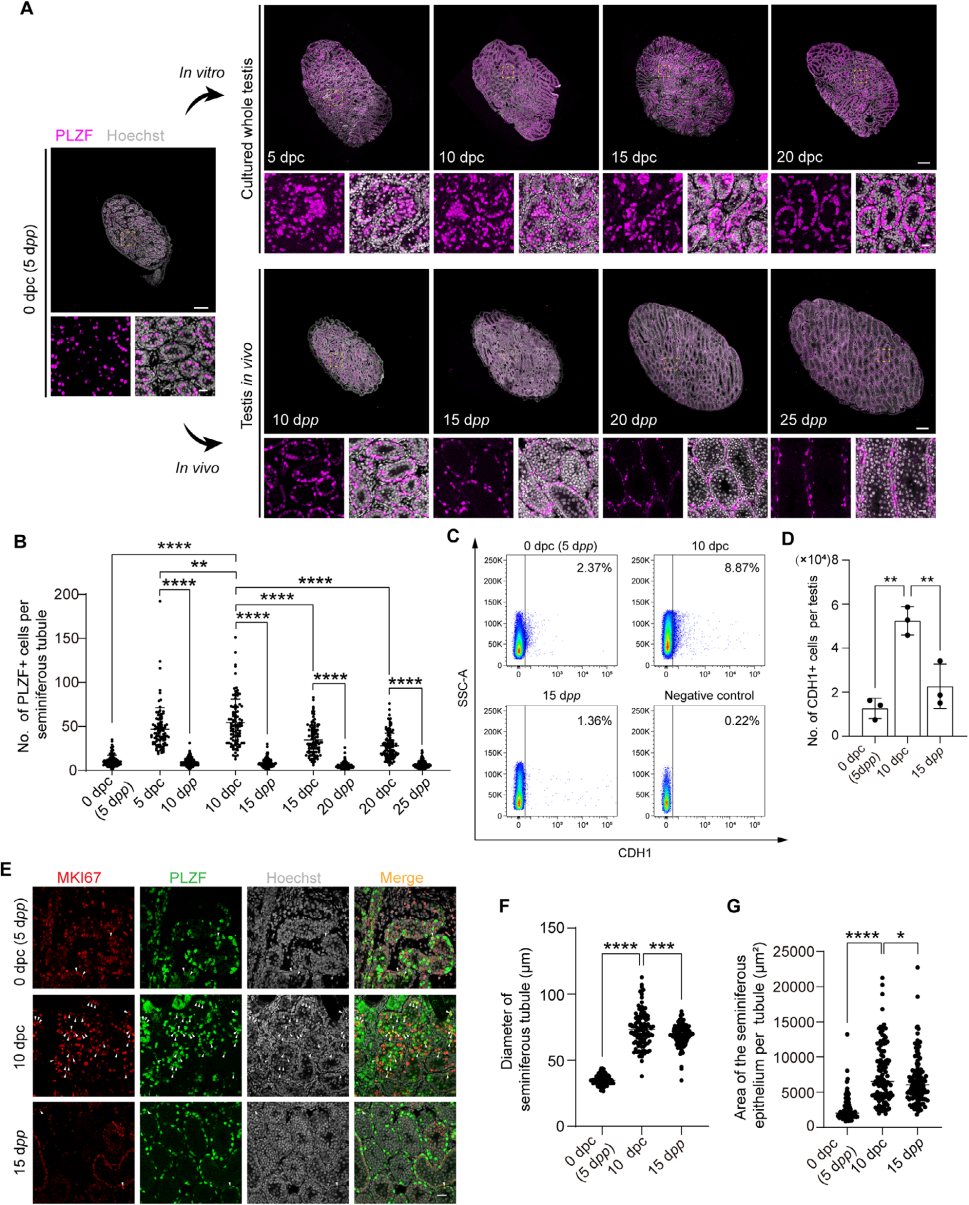
Expansion of undifferentiated spermatogonia in vitro in whole testis. A) Immunofluorescence imaging of PLZF (magenta) in cultured testicular organs and testes in vivo, with Hoechst 33342‐stained nuclei (grey). Upper panels scale bar, 200 µm; lower panels scale bar, 20 µm. The insets display high‐magnification images depicting a representative area of PLZF expression in the testes. B) Quantitative analysis of PLZF‐positive cells per seminiferous tubule in cultured testicular organs and in vivo testes. ^**^ *p* < 0.01; ^***^ *p* < 0.001; ^****^ *p* < 0.0001. Data are presented as mean value ± s.d. One‐way ANOVA. C, FACS analysis of CDH1 expression in testes at 5 d*pp*, 10 dpc, and 15 d*pp*. D) Quantification of CDH1‐positive cells per testis, analyzed via FACS. Data are means ± s.d. (n = 3), assessed by one‐way ANOVA (5 d*pp*: *p* = 0.0014; 15 d*pp*: *p* = 0.0064). E) Immunofluorescence imaging of MKI67 (red), PLZF (green), and Hoechst 33342‐stained nuclei (grey) in testes at different developmental stages. Scale bar: 20 µm. F) Measurement of the minimum diameter of seminiferous tubules using Image j. Statistical significance was assessed by one‐way ANOVA (5 d*pp*: *p* < 0.0001; 15 d*pp*: *p* = 0.0005). **G)** Measurement of the seminiferous epithelium area per tubule using Image j. Statistical significance was assessed by one‐way ANOVA (5 d*pp*: *p* < 0.0001; 15 d*pp*: *p* = 0.0394).

In order to assess the status of germ cells in mouse testes cultured with PEGDA microneedles, we measured undifferentiated spermatogonia using immunofluorescence. To this end, we first cultured the testes of 5 d*pp* mice using 20% PEGDA microneedles and measured the number of undifferentiated spermatogonia at 5‐, 10‐, 15‐, and 20 dpc. To identify time point at which the number of spermatogonia reaches its peak in the cultured testes, we assessed the quantity of undifferentiated spermatogonia marked by PLZF^[^ ^29^, ^30^ ^]^ in these samples. We compared the number of PLZF+ cells in the cultured testes with those from the testes dissected from age‐matched mice (**Figure** **2**A,B). We found that the number of PLZF+ cells at 5 dpc was five times higher than those found in the in vivo testes (10 d*pp* mice); after 10 days (corresponding to 15 d*pp*), the number was 6.5 times higher; after 15 days (corresponding to 20 d*pp*), the number was 6.4 times higher; and after 20 days (corresponding to 25 d*pp*), the number was 4.5 times higher (Figure 2A,B; Table S1, Supporting Information). We next compared the quantity of PLZF+ cells in cultured testes at 5, 10, 15, and 20 dpc with those of 5 d*pp* testes. We found that at 5 dpc, the quantity of PLZF+ cells increased 4.3‐fold, at 10 dpc a 4.9‐fold increase, at 15 dpc a 3.1‐fold increase, and at 20 dpc a 2.5‐fold increase (Figure 2A,B; Table S1, Supporting Information). Notably, the testes at 10 dpc exhibited the most pronounced growth, marking the highest rate of increase observed at any examined time point (Figure 2A,B; Table S1, Supporting Information). Together, these findings strongly suggested that a significant increase in the number of undifferentiated spermatogonia within the whole cultured testes, reaching a peak at 10 days post cultivation.

The potential of Spermatogonial Stem Cells (SSCs) is confined to a small population of undifferentiated spermatogonia.^[^ ^31^ ^]^ To measure the number of undifferentiated spermatogonia of SSCs within the undifferentiated spermatogonia, we analyzed the expression of GFRα1,^[^ ^32^, ^33^ ^]^ key SSC markers to drive SSC self‐renewal and proliferation. We found that compared to the onset of cultivation, the average number of GFRα1+ cells per tubule increased approximately by 3, 4.4, 3.7, and 2.1 times in testes at 5‐, 10‐, 15‐, and 20 dpc, respectively (Figure S2A–C, Supporting Information). The max increase at 10 dpc (Figure S2C, Supporting Information) was consistent with our PLZF staining results. On the basis of these findings, we used the 10‐day cultivation period for all subsequent experiments.

Furthermore, CDH1 is a specific marker for undifferentiated spermatogonia in mouse testes.^[^ ^34^ ^]^ FACS analysis of CDH1+ cells in mouse testes revealed a substantial increase from ≈1.2 × 10^4^ cells (2.37% of total cells) at the start of cultivation to 5.2×10^4^ cells (8.87% of total cells) after 10 days, marking a 4.3‐fold increase in CDH1+ cell number per testis (Figure 2C,D).

To assess the proliferation status of undifferentiated spermatogonia, we next examined the co‐expression of PLZF and the proliferation marker Ki‐67 in the testes (Figure 2E). We found that the ratio of PLZF and Ki‐67 double‐positive cells to PLZF+ cells significantly increased in each seminiferous tubule of the 10 dpc testes compared to the testes at 5 d*pp* and 15 d*pp* (Figure S2D, Supporting Information). Additionally, the number of PLZF and Ki‐67 double‐positive cells per seminiferous tubule was significantly higher in the 10 dpc testes than in the 5 d*pp* and 15 d*pp* mouse testes (Figure S2E, Supporting Information). These findings suggest that, compared to normal testes in vivo, undifferentiated spermatogonia in cultured testes exhibit a stronger proliferative capacity under in vitro conditions.

To further investigate the differentiation and meiosis of spermatogonia under in vitro culture conditions, we analyzed the expression of differentiation marker KIT^[^ ^35^ ^]^ and meiotic initiation marker STRA8^[^ ^36^ ^]^ in 5 d*pp*, 10 dpc, and 15 d*pp* testes. We found that in 5 d*pp* mouse testes, both differentiated spermatogonia and spermatogonia initiating meiosis are present (Figure S2F,G, Supporting Information). Interestingly, we found that KIT+ cells, as well as STRA8+ cells also showed a minor presence in 10 dpc testes (Figure S2F,G, Supporting Information). These findings suggest that despite the expansion of undifferentiated spermatogonia, the differentiation and meiotic initiation of these cells remain unchanged in 10 dpc testes.

The proliferation of spermatogonia in the testes is closely associated with the presence of Sertoli cells, which provide the necessary microenvironment for spermatogonia.^[^ ^37^ ^]^ A recent study showed that an augmentation in the number of Sertoli cells expands the niche for SSCs, thereby increasing the number of SSCs.^[^ ^38^ ^]^ Based on those findings, we chose the marker SOX9 to assess the number of Sertoli cells and tight junction marker ZO1^[^ ^39^ ^]^ to observe the formation of niches. Our immunofluorescence staining for SOX9 in 10 dpc testes showed a threefold increase in number of SOX9+ cells per seminiferous tubule compared to the 5 d*pp* testes, indicating an increase in Sertoli cells (Figure S3A,B, Supporting Information). Subsequently, ZO1 staining showed that tight junctions established in the center of the tubules at 10 dpc, with a majority of DDX4+ germ cells in 10 dpc testes locating beneath ZO1 (Figure S3C, Supporting Information). The establishment of tight junction may provide a more suitable environment for the proliferation of spermatogonia. In contrast, the tight junctions in 15 d*pp* mouse testes were primarily located at the base of the tubules. Additionally, we observed enlargement in the diameter of the seminiferous tubules and an increase in the area of the seminiferous epithelium in our system compared to 5d*pp* mouse testes (Figure 2F,G). These findings suggested that the proliferation of spermatogonia and Sertoli cells contributes to the thickening of the tubular lumen.

Collectively, our in vitro cultivation approach utilizing PEGDA microneedles successfully facilitated the proliferation of undifferentiated spermatogonia throughout the entire mouse testis, effectively establishing a whole testicular spermatogonia pool.

### 2.3 Single‐Cell RNA‐Seq Profiling Shows Cellular Changes in WTSP

**Figure 3:**
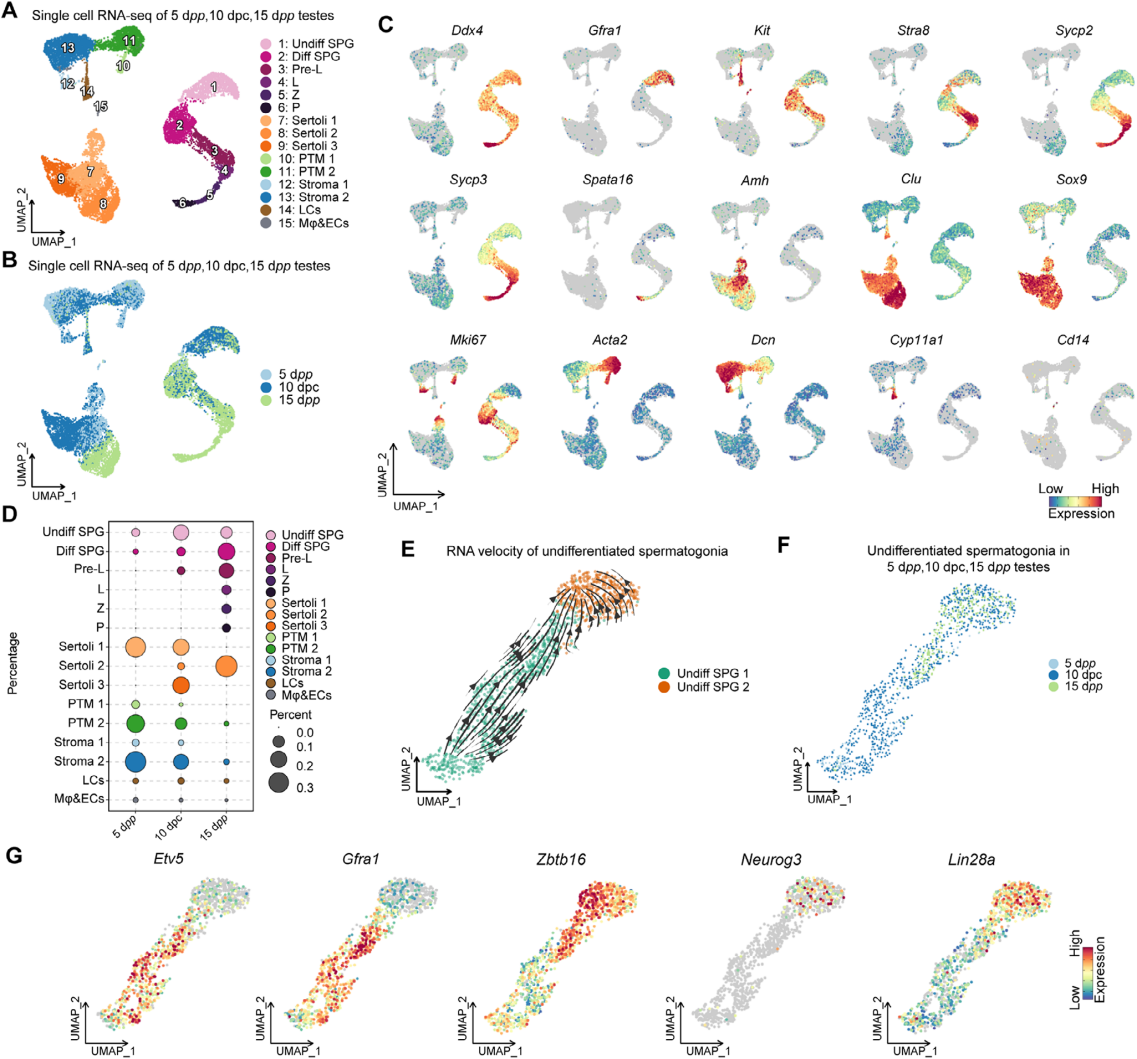
Transcriptional changes in single‐cell transcriptomes of cultured mouse testes. A) Dimensionality reduction (via UMAP) of single‐cell transcriptomic data from testes at 5 d*pp*, 15 d*pp*, and 10 dpc (n = 13,853). Each point represents a single cell, labeled with the corresponding cell category, and color‐coded based on cell type identity. B) Combined dimensionality reduction (A) of single‐cell transcriptomic data, color‐coded by different sample origins. C) Expression patterns of selected markers projected onto the UMAP plot (A). D) Circular representation of cell percentages for each cell cluster assigned to different condition samples, color‐coded according to the groups shown in panel a. E) Focused analysis (UMAP) of the Undiff SPG population, color‐coded by cell type identity, with RNA velocity annotation on the UMAP. F) UMAP of Undiff SPG population, color‐coded by different sample origins. G) UMAP plot illustrating marker genes for Undiff SPG.

To delineate the cellular landscape of the WTSP cultured in vitro, we performed 10× single‐cell RNA sequencing (scRNA‐Seq) on 5 d*pp*, 15 d*pp*, and 10 dpc testes. After quality control filtering, a total of 13 853 cells were used for further analysis. We initially integrated all scRNA‐Seq libraries using fastMNN, followed by the application of Uniform Manifold Approximation and Projection (UMAP), to process the integrated data. We identified 15 major cell types, based on the presence of known cell markers and clustering features (**Figure** **3**A,B; Figure S4A, Supporting Information).

The expression patterns of key marker genes were mapped across these identified cell types (Figure 3C; Figure S4B, Supporting Information). Groups 1‐6 represent germ cells (*Ddx4+*), consisting of undifferentiated spermatogonia (Undiff SPG, *Gfra1+*), differentiating spermatogonia (Diff SPG, *Kit+*), preleptotene (Pre‐L, *Stra8+*), leptotene (L, *Sycp2+*), zygotene (Z, *Sycp3+*), and pachytene (P, *Spata16+*). Groups 7‐15 correspond to Sertoli cells 1 (Sertoli 1, *Amh+*), Sertoli cells 2 (Sertoli 2, *Clu+*), Sertoli cells 3 (Sertoli 3, *Sox9+*), peritubular myoid cells 1 (PTM 1, *Acta2+*/*Mki67+*), peritubular myoid cells 2 (PTM 2, *Acta2+*/*Mki67‐*), Stroma cells 1 (Stroma 1, *Dcn+*/*Mki67+*), Stroma cells 2 (Stroma 2, *Dcn+*/*Mki67‐*), Leydig cells (LCs, *Cyp11a1+*), and Macrophage & Endothelial cells (Mφ&ECs, *Cd14+*).^[^ ^40^, ^41^ ^]^ Cell cycle analysis corresponded with these clusters (Figure S4C, Supporting Information). Compared to 15 d*pp*, we observed a closer resemblance in the proportions of various cell types in 10 dpc testes to those in 5 d*pp* testes, indicating the maintenance of the testicular microenvironment in vitro (Figure 3D). In 10 dpc testes, germ cells predominantly constituted undifferentiated spermatogonia, representing a proportion 3.9 times greater than in 5 d*pp* testes and 1.8 times higher than those found in 15 d*pp* testes (Figure 3D).

Our analysis identified a notable Sertoli cells subgroup (hitherto named Sertoli 3) within the 10 dpc group. This subgroup displayed distinct characteristics compared to the Sertoli cell 1 and 2. To explore the cellular state of Sertoli 3, we conducted a similarity analysis, comparing our Sertoli cells groups with those obtained by Zhao et al.^[^ ^42^ ^]^ from mouse embryos at embryonic day 17.5 (E17.5) to postnatal day 14 (PND14). These results indicated that Sertoli 1 was most similar to PND7, while Sertoli 2 and Sertoli 3 were closer to PND12‐14, suggesting a high similarity of Sertoli 3 to in vivo Sertoli cells (Figure S4D, Supporting Information). Gene Ontology (GO) term enrichment in Sertoli 3 highlighted connections to cellular responses to hypoxia and pyruvate metabolic process, common in organoid culture systems^[^ ^43^, ^44^ ^]^ (Figure S4E, Supporting Information).

To identify the cell heterogeneity of germ cells during in vitro culture, we performed a detailed subclustering analysis of germ cell cluster (Figure 3A). Through gene expression profiling, we distinguished seven distinct germ cell types (Figure S5A, Supporting Information). We found that nearly 66% of germ cells in 10 dpc testis were Undiff SPG, with minimal quantities of Diff SPG 2: and Pre‐L present (Figure S5B–D, Supporting Information). In comparison to 5 d*pp* and 15 d*pp*, almost no Diff SPG 1was observed in 10 dpc, indicating that the in vitro culture system inhibited the differentiation of undifferentiated spermatogonia into Diff SPG 1, thus enabling the undifferentiated spermatogonia to maintain their self‐renewal state (Figure S5D,E, Supporting Information).

To gain deeper insights into the subtypes of undifferentiated spermatogonia in the testis, we subclustered these cells from the 5 d*pp*, 15 d*pp*, and 10 dpc samples. Based on gene expression and clustering characteristics, we classified undifferentiated spermatogonia into Undiff SPG 1 (*Gfra1+*, *Etv5+*, *Id4+*) and Undiff SPG 2 (*Neurog3+*, *Lin28a+*, *Nanos3+*)^[^ ^45^, ^46^ ^]^ (Figure 3E–G; Figure S5F–H, Supporting Information). We then performed RNA velocity analysis to observe the developmental trajectory of undifferentiated spermatogonia. (Figure 3E). Our cellular clustering map showed that Undiff SPG 1 was predominant in the 5 d*pp* testis, whereas Undiff SPG 2 was more prevalent in the 15 d*pp* testis (Figure S5F, Supporting Information). Intriguingly, in 10 dpc testes, we found that the proportions of both Undiff SPG 1 and Undiff SPG 2 increased, suggesting that both subtypes effective proliferated during in vitro expansion (Figure 3F; Figure S5F, Supporting Information).

Together, these findings suggested that our WTSP maintains the testicular microenvironment of 5 d*pp* testis, and critically, facilitates the proliferation of undifferentiated spermatogonia.

### 2.4 Role of EPHA2 in Regulating Undifferentiated Spermatogonia Proliferation and Meiosis Initiation in WTSP

**Figure 4:**
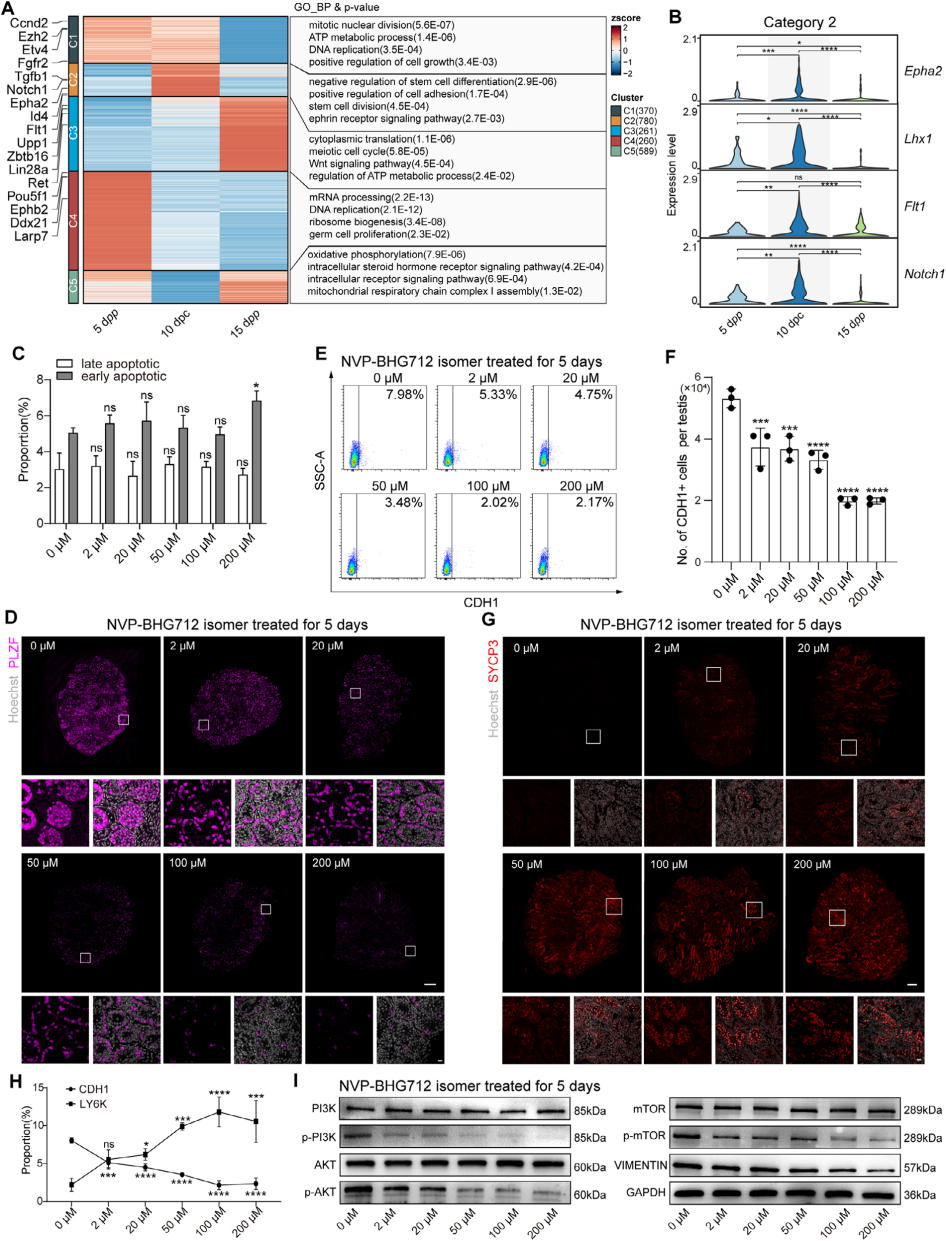
Inhibition of EPHA reduces proliferation of undifferentiated spermatogonia and initiates meiosis in WTSP. A) Hierarchical clustering of differentially expressed genes in undifferentiated spermatogonia in different samples (5 d*pp*, 10 dpc, and 15 d*pp*), along with corresponding GO enrichment analysis results for different categories. B) Violin plots depicting the expression levels of category 2 genes related to Undiff SPG proliferation in different samples. C) FACS‐detected percentages of late (Annexin V+/PI+) and early (Annexin V+/PI‐) apoptotic cells in 5 dpc testes treated with varying concentrations of NVP BHG712 isomer. Statistical significance was assessed by one‐way ANOVA. P value for comparison between different concentrations and the control group without NVP BHG712 isomer, late apoptotic: 2 µm = 0.9963; 20 µm = 0.9544; 50 µm = 0.9758; 100 mm = 0.9996; 200 µm = 0.9580; early apoptotic: 2 µm = 0.7371; 20 µm = 0.5624; 50 µm = 0.9673; 100Μm = 0.9997; 200 µm = 0.0153. Data are presented as mean values ± s.d., n = 3. D) Immunofluorescence imaging of PLZF (magenta) in 5 dpc testes treated with different concentrations of NVP BHG712 isomer. Nuclei stained with Hoechst 33342 (grey). Upper panels scale bar, 200 µm; lower panels scale bar, 20 µm. The insets display high‐magnification images depicting a representative area of PLZF expression in the testes. E) FACS analysis showing CDH1 expression in 5 dpc testes treated with varying concentrations of NVP BHG712 isomer. F) Quantification of CDH1‐positive cells per 5 dpc testis, following treatment with different concentrations of NVP BHG712 isomer, as analyzed by flow cytometry. Statistical significance was assessed by one‐way ANOVA (2 µm: *p* = 0.0007; 20 µm: *p* = 0.0005; 50 µm: *p* < 0.0001; 100 µm: *p* < 0.0001; 200 µm: *p* < 0.0001). Data represent means ± s.d. (n = 3). G) Immunofluorescence detection of SYCP3 (red) and the nuclei was stained by Hoechst 33342 (grey) in 5 dpc testes after treatment with NVP BHG712 isomer of varying concentrations. Scale bars: 200 µm (upper panels), 20 µm (lower panels). The insets display high‐magnification images depicting a representative area of SYCP3 expression in the testes. H) Percentages of CDH1, LY6K expression in 5 dpc testes after treatment with NVP BHG712 isomer of varying concentrations, detected by FACS. P value for comparison between different concentrations and the control group without NVP BHG712 isomer, CDH1: 2 µm = 0.0001; 20 µm < 0.0001; 50 µm < 0.0001; 100 µm < 0.0001; 200 µm < 0.0001, LY6K: 2 µm = 0.0819; 20 µm = 0.0343; 50 µm = 0.0003; 100 µm < 0.0001; 200 µm = 0.0001. Data are presented as mean values ± s.d., n = 3. Statistical significance was assessed by one‐way ANOVA. I) Western blot analysis of PI3K, p‐PI3K, AKT, p‐AKT, mTOR, p‐mTOR, and VIMENTIN in 5 dpc testes treated with varying concentrations of NVP BHG712 isomer.

To investigate the genes that drive undifferentiated spermatogonia growth in WTSP, we analyzed differential gene expression (DEG) of undifferentiated spermatogonia at 5 d*pp*, 10 dpc, and 15 d*pp*. We categorized DEG into five distinct categories using K‐means hierarchical clustering. Category 1 (C1): Genes activated at 5 d*pp* and 10 dpc, decreasing at 15d*pp*. GO enrichment analysis unveiled that C1 genes are implicated in mitotic nuclear division and DNA replication. C2: Genes peaking at 10 dpc, reduced at 5 d*pp* and 15 d*pp*, which contributed to stem cell division and ephrin (EPH) receptor signaling pathway, potentially associated with the proliferation of undifferentiated spermatogonia in the testes at 10 dpc. C3: Genes consistently decreased at 5 d*pp* and 10 dpc, activating at 15 d*pp*. These genes were associated with the meiotic cell cycle and WNT signaling pathway. C4: Genes dominated at 5 d*pp*, tapering thereafter, mainly participating in DNA replication and germ cell proliferation. C5: Genes pronounced at 5 d*pp* and 15 d*pp*, subdued at 10 dpc, primarily associated with oxidative phosphorylation pathways (**Figure** **4**A,B; Figure S6A, Supporting Information). The EPH receptors *Epha2* and *Ephb2*, as key genes, are known for maintaining SSC self‐renewal.^[^ ^47^, ^48^ ^]^

We found that *Ephb2* (category C4) expressed at a high level at 5 d*pp*, suggesting that it plays a critical role in the early proliferation of SSCs in mice (Figure 4A; Figure S6A, Supporting Information). Although *Ephb2* continued to be expressed at 10 dpc, we observed a shift in expression in 10 dpc testes, where the expression of Epha2 (category C2), was significantly elevated (Figure 4A,B).

To elucidate the role of EPHA2 in spermatogonia proliferation, we incorporated the EPH kinase inhibitor^[^ ^49^ ^]^ called NVP‐BHG712 isomer into our culture system. We found that this inhibitor did not increase TUNEL+ cells in the testes (Figure S6B, Supporting Information). We next used Annexin V/PI staining to detect early (Annexin V+/PI‐) and late (Annexin V+/PI+) apoptotic cells across the entire testis. We found that at concentrations below 100 µm NVP‐BHG712 isomer showed little effect on the occurrence of both early and late stages of cell apoptosis in the 5 dpc testis (Figure 4C; Figure S6C, Supporting Information).

Next, we assessed the effect of inhibiting EPHA2 on cultured testicular tissues using immunofluorescence and FACS analysis. We found that the numbers of PLZF+ cells gradually decreased with increasing concentrations of NVP‐BHG712 isomer (Figure 4D). CDH1 is a specific marker for undifferentiated spermatogonia in mouse testes.^[^ ^34^ ^]^ Our FACS results showed that 5 dpc testes significantly decreased in CDH1+ cells at 2 µm inhibitor concentration, with a reduction of 75% at concentrations above 100 µm, compared to the group without NVP‐BHG712 isomer (0 µm, with DMSO added) (Figure 4E,H). Furthermore, when we added NVP‐BHG712 isomer, we found that total undifferentiated spermatogonia counts in 5 dpc testes decreased as determined by FACS counting of CDH1+ cells per testis (Figure 4F). These results indicated that inhibiting EPHA2 suppresses spermatogonia proliferation.

Simultaneously, as the inhibitor concentration increased, the number of SYCP3+ spermatocytes increased (Figure 4G). Further FACS analysis for LY6K, a spermatocyte‐specific membrane protein,^[^ ^50^ ^]^ showed that the number of spermatocytes increased as a function of the inhibitor (Figure 4H; Figure S6D, Supporting Information). These findings implied that EPHA2 inhibition fosters the formation of spermatocytes.

The EPHA2 receptor is known to stimulate the proliferation of tumor cells by activating downstream proteins, including VIMENTIN, through the phosphorylation of proteins in the PI3K‐AKT‐mTOR pathway.^[^ ^51^ ^]^ To investigate whether EPHA2 plays a role in spermatogonia proliferation via this pathway, we assessed the protein expression of the PI3K‐AKT‐mTOR pathway using western blot analysis. We found that while the overall levels of PI3K, AKT, and mTOR remained constant, their phosphorylation levels, as well as the expression of downstream VIMENTIN, progressively decreased with increasing concentrations of the EPHA2 inhibitor (Figure 4I). Together, these findings indicated that EPHA2 inhibition results in reduced phosphorylation in the PI3K‐AKT‐mTOR pathway and a subsequent decrease in VIMENTIN expression, ultimately suppressing the proliferation of undifferentiated spermatogonia in WTSP.

### 2.5 High Spermatogenic Capacity of Undifferentiated Spermatogonia in WTSP

**Figure 5:**
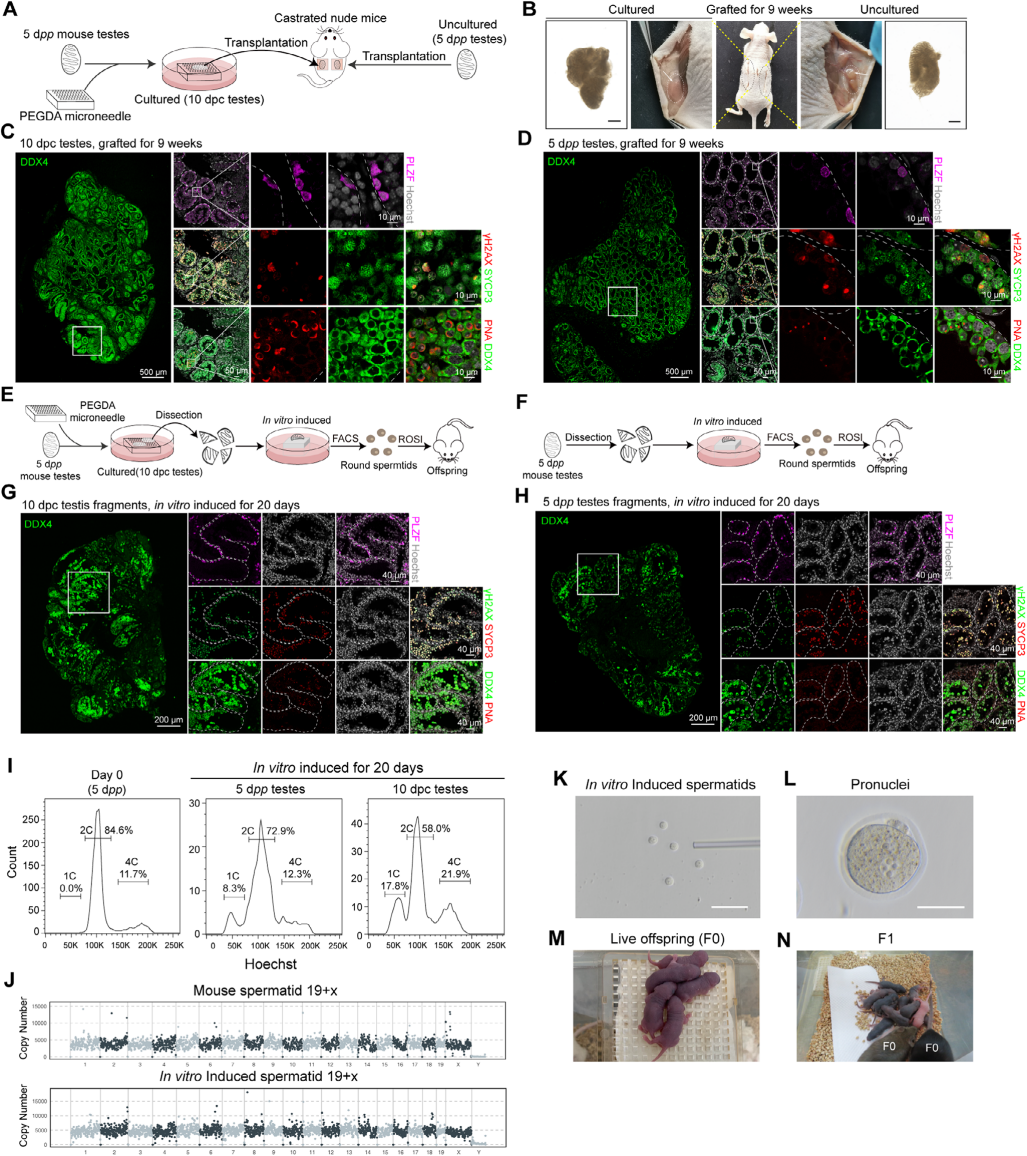
WTSP has the ability to regenerate spermatogenesis both in vivo and in vitro. A,B) Schematic of the transplantation experiment. Scale bar, 1 cm. C,D) Immunofluorescence detection of germ cells (DDX4), undifferentiated spermatogonia (PLZF), spermatocytes (γH2AX/SYCP3), spermatids (DDX4/PNA) in 10 dpc testes and 5 d*pp* testes after grafted for 9 weeks. The insets display high‐magnification images depicting a representative area (from serial paraffin sections) of PLZF, γH2AX/SYCP3 and DDX4/PNA expression in the testes. E,F) Schematic of the in vitro spermatogenesis experiments using WTSP and 5 d*pp* testes. G,H) Immunofluorescence imaging of germ cells (DDX4), undifferentiated spermatogonia (PLZF), spermatocytes (γH2AX/SYCP3), and spermatids (DDX4/PNA) in 10 dpc and 5 d*pp* testis tissues after 20 days of in vitro induced. The insets display high‐magnification images depicting a representative area (from serial paraffin sections) of PLZF, γH2AX/SYCP3 and DDX4/PNA expression in the testes. I) Flow cytometry analysis of DNA content distribution in cells from in vitro induced of 5 d*pp* and 10 dpc testes, denoting haploid (1C), diploid (2C), and tetraploid (4C) cells. J) Genomic DNA sequencing of in vitro‐derived single spermatid and in vivo mouse spermatid sorted by FACS. K) Isolated spermatids from 10 dpc testis tissues differentiated in vitro for 20 days. Scale bar, 25 µm. L) Embryos at the pronuclear stage with two pronuclei. Scale bar, 50 µm. M) Offspring of round spermatid injection (ROSI) using round spermatids derived from 10 dpc testis. Scale bar, 50 µm. N) Fertility assessment of offspring produced from in vitro‐induced round spermatids.

To investigate the spermatogenic capacity of germ cells in WTSP, we transplanted 10 dpc testes under the skin on the left dorsal side of castrated adult nude mice. Testes from 5 d*pp* mice were transplanted on the right dorsal side of the same mice, and used for control experiments (**Figure** **5**A,B). Nine weeks post transplantation, we assessed spermatogenesis in the transplanted testes using immunofluorescence analysis. Both 10 dpc and 5 d*pp* transplanted testes exhibited spermatogenesis (Figure 5C,D). We found that the seminiferous epithelium in the transplanted WTSP contained a higher number of SYCP3/γH2AX double‐positive spermatocytes and PNA+ spermatids within the tubules, compared to the 5 d*pp* transplanted testes (Figure 5C,D; Figure S7A,B, Supporting Information). By performing immunofluorescence counting across multiple transplants, we observed that although the number of spermatocytes and spermatids increased in the transplanted 10 dpc testes, the proportion of seminiferous tubules containing SYCP3⁺ spermatocytes or PNA⁺ spermatids showed no significant difference between the 10 dpc and 5 d*pp* groups (Figure S7C,D, Supporting Information).

Together, our findings clearly demonstrated that in vivo transplantation of WTSP resulted in the production of more spermatocytes and spermatids.

### 2.6 Healthy Offspring from Spermatids Derived from WTSP In Vitro

To mitigate the risk of malignant contamination in in vivo transplantation for cancer patients,^[^ ^7^ ^]^ we shifted our focus to in vitro spermatogenesis as a potential solution for this issue. To induce spermatid formation in 5 d*pp* and 10 dpc testes, we gently divided the testes into 4–5 pieces and placed them on agarose gel using the in vitro spermatogenesis culture medium (composition details provided in the Experimental Section) (Figure 5E,F; Figure S7E, Supporting Information). Following induction of spermatid formation for 20 days, our immunofluorescence analysis showed that 10 dpc‐induced group had a significantly higher number of PNA+ spermatids and SYCP3+/γH2AX+ spermatocytes in 10 dpc‐induced group compared to the 5 d*pp*‐induced group (Figure 5G,H; Figure S7F,G, Supporting Information).

Subsequent FACS analysis showed that the proportion of haploid cells in 10 dpc‐induced group was 17.8%, over twice that in the 5 d*pp*‐induced group (Figure 5I). When we performed chromosome spreading experiments, we found that spermatocytes in the 10 dpc‐induced group underwent leptotene, zygotene, pachytene, and diplotene stages during meiosis (Figure S7H, Supporting Information). We also found that proliferative undifferentiated spermatogonia (GFRα1+/KI67+), differentiated spermatogonia (KIT+), and preleptotene spermatocytes (STRA8+) were simultaneously present within the same seminiferous tubules (Figure S7I, Supporting Information). These findings clearly demonstrated that spermatogenesis is sustained in vitro.

To verify the genomic integrity of in vitro induced spermatids, we conducted single‐cell whole‐genome sequencing on FACS‐purified spermatids from the 10 dpc‐induced group to analyze copy number variations (CNVs). Of the induced cells from the 10 dpc testes, seven single haploid cells were analyzed, with four displaying a normal haploid genome, one cell was haploid with chromosomal deletions, and two were diploid (Figure 5J). To assess the functionality of spermatids derived from in vitro induction, we performed round spermatid injection (ROSI) experiments. The fertilization rates observed were 47.9% for spermatids from 10 dpc‐induced group, and 60% for those from 5 d*pp*‐induced group. Subsequent offspring production rates were 26.1% and 16.7%, respectively (Figure 5K–M; Table S2, Supporting Information). Importantly, the progeny from 10 dpc‐induced spermatids exhibited normal weight growth and fertility, comparable to the control group (Figure 5N; Figure S7J,K, Supporting Information).

Together, these findings demonstrated that germ cells within WTSP are capable of undergoing meiosis and to produce functional spermatids, thus underscoring the robust spermatogenic potential of cells cultivated within this system.

### 2.7 Enhanced Spermatogonia Proliferation in Cultured Human Gonadal Ridges

**Figure 6:**
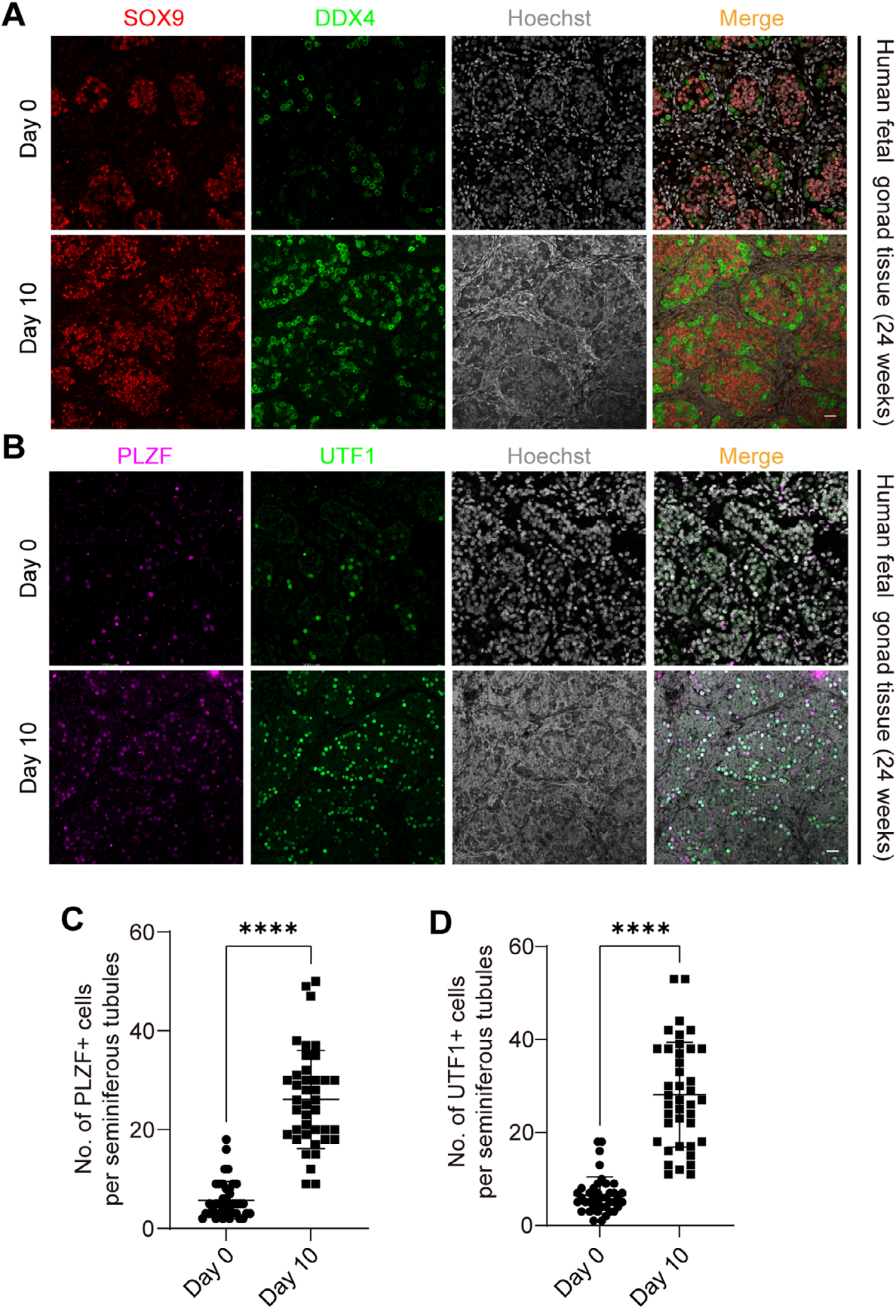
Enhanced Spermatogonia Proliferation in Cultured Human Gonadal Ridges. A) Immunofluorescence imaging of SOX9 (red), DDX4 (green) in cultured testicular organs, with Hoechst 33342‐stained nuclei (grey). Scale bar, 20 µm. B) Immunofluorescence imaging of PLZF (magenta), UTF1 (green) in cultured testicular organs, with Hoechst 33342‐stained nuclei (grey). Scale bar, 20 µm. C,D) Quantitative analysis of PLZF‐positive cells and UTF1‐positive per seminiferous tubule in cultured testicular organs. *p* < 0.0001. Data are presented as mean value ± s.d. Two‐tailed Student's t‐tests.

Germ cells in late‐gestation gonadal ridges closely resemble spermatogonia found in postnatal and adult testes.^[^ ^22^ ^]^ To evaluate the applicability of our culture system to human testicular tissue, we cultured gonads harvested from three aborted human male fetuses (24–25 weeks) using the PEGDA microneedle. UTF1 is a marker for precursor spermatogonia and spermatogonial stem cells of human,^[^ ^52^, ^53^ ^]^ while PLZF specifically marks undifferentiated spermatogonia.^[^ ^54^ ^]^ By assessing these markers, we quantified the proliferation of different spermatogonial populations within the cultured tissue. After 10 days of culture, we observed a more than fourfold increase in UTF1‐positive spermatogonia and PLZF‐positive spermatogonia per seminiferous tubule, indicating robust spermatogonial proliferation under these conditions (**Figure** **6**A–D). These findings suggest that our microneedle‐based culture system effectively supports the expansion of human spermatogonia, underscoring its potential for fertility preservation applications.

## 3 Discussion

Traditional 3D culture systems often face challenges in supporting central cell survival,^[^ ^55^ ^]^ particularly in the context of in vitro testicular tissue culture.^[^ ^11^ ^]^ In summary, we developed a PEGDA microneedle‐based WTSP culture system that significantly increases nutrient distribution and supports both spermatogonia self‐renewal as well as proliferation in mouse testis. The WTSP demonstrated higher efficiency in spermatid formation, both in vivo and in vitro, which proves the functionality of spermatogonia and somatic cells within the WTSP.

Currently used technologies face significant challenges in preserving fertility for prepubertal boys with cancer, due to the limited production of viable sperm in vitro and the substantial risks associated with testicular tissue transplantation. Our culture system, however, shows promise in overcoming these hurdles by generating a higher quantity of spermatids. Our WTSP system improves the efficiency of spermatid formation, as evidenced by successful subcutaneous transplantation in vivo and in vitro induction of meiosis. The observed increase in the efficiency of spermatid production strongly suggests that cells derived from our WTSP system constitute an effective basis for creating functional spermatids in assisted reproductive technology procedures, presenting a groundbreaking method for fertility preservation in prepubertal boys.

Notably, due to the difficulty in obtaining prepubertal testicular samples, we employed human gonadal ridge tissue for cultivation. Spermatogonia in the human gonadal ridge tissue exhibited remarkable proliferation after cultivation, highlighting the potential of this system to establish a supportive microenvironment for germ cell expansion. Although fetal gonadal ridge tissue and prepubertal testicular tissue are not developmentally identical, spermatogonia within late‐stage fetal gonads exhibit transcriptional and developmental characteristics that closely resemble those of postnatal spermatogonia. The robust proliferation observed in our culture system indicates the presence of conserved regulatory features, supporting the potential utility of this model in advancing fertility preservation strategies. Previous studies have established organotypic and organoid culture systems using prepubertal testicular tissue, demonstrating germ cell differentiation at various stages, different states of somatic cells, and certain endocrine functions.^[^ ^56^, ^57^, ^58^, ^59^ ^]^ In these systems, a declining trend in spermatogonial cells over time has also been observed, which may limit the efficiency of germ cell differentiation.^[^ ^57^ ^]^ In contrast, our system promotes significant spermatogonial proliferation, which may provide valuable support for future strategies aiming to induce spermatogenesis in vitro.

In vivo study of germ cells poses significant challenges, stemming from the testes’ complex structure and the diverse array of cell types involved. SSCs are pivotal for ongoing spermatogenesis and are regulated through interactions with Sertoli cells and peritubular myoid cells.^[^ ^60^ ^]^ However, the rarity of SSCs and their diverse developmental stages within the testicular cell population have made it challenging to study niche factors crucial for SSC self‐renewal and differentiation.^[^ ^61^ ^]^ Our system offers a substantial advantage by providing a 3D tissue culture environment for examining the developmental states of germ cells. So far, only a limited number of studies have performed transcriptomic profiling on in vitro cultured testicular tissues to explore similarities and differences between in vitro and in vivo spermatogenesis.^[^ ^62^ ^]^ Our study adds to this growing field by providing a 3D culture system combined with single‐cell resolution, enabling precise characterization of germ and somatic cell states under defined culture conditions. This platform offers a valuable resource for further dissecting the dynamics of germ cell development and testicular niche interactions in vitro.

The EPHA pathway was previously shown to be critically involved in the regulation of SSCs.^[^ ^47^ ^]^ In agreement, our findings highlight the role of EPHA2 in promoting spermatogonia proliferation. The EFNA ligand family^[^ ^63^ ^]^ of EPHA2 in peritubular myoid and Sertoli cells exhibits higher expression levels at 10 dpc, suggesting a significant role of EFNA‐EPHA2 interactions in spermatogonia proliferation. Our results clearly demonstrate that EPHA2 plays a critical function in activating the PI3K‐AKT‐mTOR pathway during spermatogonia proliferation, as shown by using an EPHA2 receptor inhibitor in vitro.

While our study provides new methods for fertility preservation in prepubertal boys and for researching self‐renewal and meiosis of spermatogonia, a number of limitations remain. First, the dense insertion of PEGDA microneedles into the entire testis restricts the expansion of the seminiferous tubule lumen, limiting the proliferation of spermatogonia and further spermatogenesis in WTSP. Future efforts should focus on selecting appropriate biological materials and designing optimal shapes for testis culture, aiming to enhance the yield of functional spermatids further. Additionally, further studies on the genetic integrity and long‐term health outcomes of offspring derived from WTSP‐induced spermatogenesis are crucial.

In conclusion, the WTSP system represents a significant advancement in the field of fertility preservation. Its ability to support spermatogonia proliferation opens new possibilities for individuals facing the risk of infertility, particularly in pediatric oncology settings. Continued research and refinement of this system hold the promise of expanding fertility preservation options and improving outcomes for those in need.

## 4 Experimental Section

### Ethics Statement

All animal study protocols were approved by the Institutional Animal Care and Use Committees (IACUC) of Nanjing Medical University (ethics approval no.IACUC‐2009002). Human fetal gonads were obtained from aborted fetuses with the approval of the Ethical Committee of Nanjing Maternity and Child Health Care Hospital (Permission Number (2017)68). Written informed consents were obtained from all participants after they had independently and legally chosen to terminate their pregnancy. No financial incentives were provided. The study complied with all local legal regulations and institutional ethical requirements. Ethical considerations related to fetal development were carefully addressed, and participants’ confidentiality and rights were fully protected.

### Mice

C57BL/6J and ICR strain mice were housed in an SPF animal facility under a 12 h–12 h light–dark cycle, and the room with rooms maintained at a temperature of 22 °C and 45% humidity.

### Fabrication of Microneedles (MNs)

2‐Hydroxy‐2‐methy ipropiophenone (HMPP), poly (ethylene glycol) diacrylate (PEGDA, Mn 700), and polyethylene glycol (PEG) were purchased from Sigma–Aldrich Co. Rhodamine B was purchased from Hefei BOSF. The pre‐hydrogel solution with 20% (v/v) PEGDA, 20% (v/v) PEG, and 1% (v/v) HMPP was injected into microneedle molds and vacuumed until no bubbles. UV lamp exposure was utilized to polymerize the hydrogel, after which the microneedles were carefully peeled off from the molds.

### Permeation Depth Test

Microneedles loaded with rhodamine B were inserted under the agarose. Permeation depth of rhodamine B from the tip of the needle upward was recorded at 10, 20, 30, and 40 min.

### Biocompatibility Test In Vitro

NIH‐3T3 cells were co‐cultured with microneedles for 3 days. Cell growth and viability were assessed using microscopy and MTT assays.

### Microneedle‐Based Whole Testes Culture System

The 20% PEGDA MNs were pre‐soaked in culture medium for at least two days. The culture medium composition was based on StemPro®‐34 SFM (Gibco, 10639011), modified as described previously,^[^ ^64^ ^]^ supplemented with 0.5% BSA (Sigama A3803), 1% FBS (Invitrogen 16141061), 2 mm GlutaMAX (Gibco 35050061), 0.1 mm NEAA (Gibco 11140050), 1 mm Sodium Pyruvate (Gibco 11360070), 1 ml ml^−1^ DL‐ Lactate (Sigma L4263), 0.1 mm 2‐mercaptoethanol (Gibco 21985023), 25 µg mL^−1^ Insulin (Sigma 11376497001), 100 µg mL^−1^ Transferin (sigma T1147), 30 nm Sodium Selenite (Sigma S1382), 60 0ng mL^−1^ Progesterone (Sigma P8783), 300 ng mL^−1^ Estrogen (Sigma E2758), 5 mg mL^−1^ D‐(+)‐Glucose (Sigma G7021), 10 µg mL^−1^ Biotin (Sigma B4501), 17.6 µg mL^−1^ Ascorbic acid (Sigma A4544), 50 ng mL^−1^ EGF (R&D, 236‐EG‐01M), 10 ng mL^−1^ bFGF (R&D, 233‐FB‐500), 80 ng mL^−1^ GDNF (R&D, 512‐GF‐050). For the in vitro inhibition of EPHA2 experiment, different concentrations of NVP‐BHG712 isomer (MCE HY‐13258) were added to the culture medium, with the control group (0 µm) lacking NVP‐BHG712 isomer.

The testes of 5dpp pup mice were decapsulated, and gently positioned on the tip of a PEGDA microneedle soaked in culture medium. The amount of medium was adjusted to ensure that the liquid level did not exceed the hydrogel substrate. Whole testes were cultured in an atmosphere of 5% CO_2_ in air at 34.0 °C, with media changed daily.

### Histology and Immunofluorescence (IF) Analysis

Testicular tissues were collected and fixed with 4% (v/v) PFA (Sigma P6148), then dehydrated, paraffin embedded and sectioned for further histologic analysis. Paraffin blocks were sectioned at 5 µm and dried overnight at 65 °C before deparaffinization, rehydration, and epitope retrieval. Non‐specific antibody binding was blocked with 5% BSA (diluted in PBS) for 2 h at room temperature. Primary antibodies were incubated overnight at 4 °C in blocking buffer, and then the sections were rinsed with PBS three times for 10 min each time and incubated with secondary antibodies diluted in blocking buffer at 37 °C for 2 h, before the sections were washed with PBS 3 times for 10 min each time. The following primary antibodies were used: normal mouse IgG (1:100, Santa Cruz, sc‐2025), normal rabbit IgG (1:100, Santa Cruz, sc‐2027), normal goat IgG: (1:100, Santa Cruz, sc‐2028), DDX4 (1:500, Abcam, ab27591), SOX9 (1:500, Millipore, AB5535), γH2AX (1:500, Abcam, Ab26350), ZO1 (1:200; Thermo Fisher, 617300), GFRα1 (1:500; R&D systems, AF560), PLZF (1:500; R&D systems, AF2944), CKIT (1:200; R&D systems, AF1356), KI67 (1:500, Abcam, Ab16667), SYCP3 (1:200, Abcam, ab97672). The following secondary antibodies were used: Donkey anti‐Rabbit IgG (1:500, Thermo Fisher Scientific, A‐21206), Donkey anti‐Mouse IgG (1:500, Thermo Fisher Scientific, A‐31570), Donkey anti‐Goat IgG (1:500, Thermo Fisher Scientific, A‐21447). All sections were counterstained using Hoechst 33342. Images were acquired on a LSM800 confocal microscope (Carl Zeiss) using ZEN 2012 (blue edition).

### TUNEL Assay

Apoptosis was measured using the TUNEL assay kit (Vazyme, A113) according to the manufacturer's instructions. Testicular tissue sections were hydrated and then soaked and rinsed in PBS. Sections were incubated in 20 µg mL^−1^ Protease K for 20 min at room temperature, then washed three times with 1X PBS for 3 min each time, following incubated with DNA labelling solution prepared according to the instructions for 1 h at 37 °C in a dark humidified incubator. The labeled sections were washed twice with 1x PBS for 5 min each time, followed by counterstaining with freshly prepared Hoechst 33342 in 1 × PBS for 5 min at room temperature in the dark, and then washed three times with 1 × PBS for 5 min each time. The samples were kept moist with 20% glycerol prepared in 1 × PBS and stored at 4 °C in the dark. Imaging was done using a confocal microscope (Zeiss), and TUNEL‐positive cells were quantified using ImageJ.

### Flow Cytometry Analysis

Cultured or in vivo testes were dissociated into single cells by incubating with 0.25% Trypsin‐EDTA (Gibco, 25200072) at 37 °C for 5 min. DMEM (Gibco, 11995‐065) containing 10% FBS (Gibco, 10099141C), was added to inactivate trypsin. Cells were collected by centrifugation and incubated with PE anti‐mouse/human CD324 (CDH1) Antibody (Biolegend, 147303) or PE anti‐mouse Ly6K Antibody (Biolegend, 151304) in PBS for 15 min at room temperature. For ploidy analysis, single‐cell suspensions were stained with 10 mg mL^−1^ Hoechst 33342 for 20 min and washed three times with PBS. For counting the number of CDH1, FACS was performed on a FACSAria Fusion SORP (BD Biosciences), with all cells from a single testis recorded.

### Chromium Next GEM Single Cell RNA 3’library Construction

The 10X genomics user guide was followed for all steps. The Chromium Next GEM Single Cell 3ʹ GEM, Library & Gel Bead Kit v3.1 (10X Genomics) was used. In brief, all single‐cell suspensions and reagents were mixed and loaded into the chip to generate GEMs using the Chromium Controller. GEM‐RT incubation was performed in the droplets. Single‐stranded cDNA was recovered through demulsification and dynabeads cleanup. After PCR, pre‐amplified cDNA products were subjected to SPRIselect and further library construction. Finally, the libraries were quantified, quality‐controlled, and sequenced on an Illumina NovaSeq 6000 sequencer at the Nanjing Jiangbei New Area Biopharmaceutical public service platform (Nanjing, China).

### Processing of Single Cell RNA‐Seq Data

Sequencing data were processed using the 10x Genomics Cell Ranger (v.6.0.2) program to align (using the mm10 reference genome), filter, and calculate the unique molecular identifier (UMI) for each sample using the “‐includeintrons” parameter. The scDblFinder (v1.10.0) algorithm was first applied to each sample to predict potential twin default parameters at the default parameters.^[^ ^65^ ^]^ All cells labeled as twins were removed in the downstream analysis. Cells with >1500 expressed genes, UMI >5500, <20% of reads mapping to the mitochondrial genome, and <30% of reads mapping to the ribosomal genome were retained. The three samples were then integrated and analyzed by fastMMN,^[^ ^66^ ^]^ and U‐MAP and clustering analyses were performed on the combined dataset using the top 2500 highly variable genes and 1‐40 PCs. Cell dynamics were analyzed using the scVelo (v0.2.4) package.^[^ ^67^ ^]^ The similarity between different single‐cell RNA sequencing datasets was predicted using the KNN method. The hierarchical clustering of the heatmap is built using the K‐means method, with k set to 5.

### Differential Gene Expression Analysis and Enrichment Analysis

The FindMarkers function of the Seurat pipeline was used to compare cells from one cell type with cells from all other cell types to find differentially expressed genes for that cell type.^[^ ^68^ ^]^ The parameters test wa used for each comparison. The resulting p‐value is adjusted using the BH technique, with use = “Wilcox”, min.pct = 0.1, and logfc.threshold = log2 (1.5). Using the enricher feature of the ClusterProfiler, marker genes for each cell type were filtered (p_val_adj<0.05) and utilized to conduct a GO study package (v4.4.4) with parameters minGSSize = 10, maxGSSize = 500, and adjust method = “BH” using the Biological Process (BP) annotation in the org.Hs.eg.db (v3.13.0) package.^[^ ^69^ ^]^ Biological Process (BP) terms were filtered with a p_adjustCutoff < 0.05, and the p‐values for these terms were plotted.

### Apoptosis Assays

The percentage of apoptotic cells after treated with EPHA inhibitors was quantified using the Annexin V‐Alexa Fluor647/PI Apoptosis Detection Kit (YEASEN 40304ES60). Testes were digested into single cells (see Flow Cytometry Analysis). 5 µL of Annexin V‐Alexa Fluor 647 and 10 µL of PI solution were added to each sample tube contains 500 µL of the resuspended cells, followed by co‐incubation for 15 min at room temperature in the dark. 400ul of 1×Binding Buffer was added to each tube of samples, which were then analysed by flow cytometry (BDVerse). The different phases of apoptosis were defined as:1) live cells (Annexin V‐ PI‐), 2) early apoptotic (Annexin V+ PI‐), 3) late apoptotic (Annexin V+ PI+), 4) necrotic cells (Annexin V‐ PI+). The results were further collated and plotted using Flowjo software. The same experiment was repeated three times independently.

### Protein Samples and Western Blot Analysis

Total protein from cultured testes was extracted with RIPA buffer (Beyotime, P0013C) supplemented with 1% protease Inhibitor Cocktail(Selleck, B14002)and 2% phosphatase inhibitors (Beyotime, P1081). The samples were incubated for 30 min on ice, vortexed every 15min, and then clarified by centrifuging for 30 min at 12 000 rpm at 4 °C. The protein samples were added with loading (5×), 95 °C heated for 10 min. Proteins were separated by SDS–PAGE and transferred onto polyvinylidene difluoride membrane. (BIO‐RAD, 1620177). The membrane was blocked with 5% BSA in tris‐buffered saline solution (TBS) for 2 h at room temperature, and then incubated overnight at 4 °C with the following primary antibodies: anti‐mTOR (1:1,000, CST, 2983T), anti‐p‐mTOR (1:1,000, CST, 5536T), anti‐PI3K (1:1,000, CST, 4257T) and anti‐p‐PI3K (1:1,000, Abcam, ab182651), anti‐AKT (1:5,000, CST, 4691), anti‐p‐AKT (1:2,000, Proteintech, 66444‐1‐Ig), Vimentin (1:1,000, CST, 5741S), GAPDH (1:5000, Proteintech, 60004‐1‐Ig). The next day, the membranes were washed three times within TBS containing 0.1% (v/v) Tween‐20 (TBST) for 10 mins each time and then incubated at room temperature for 2 h with the secondary antibodies: goat anti‐rabbit IgG (1:1,000, Beyotime, A0208) and goat anti‐mouse IgG (1:1,000, Beyotime, A0216). After rinsing with TBST, the signals from the detected proteins were visualized using the SuperSignal West Femto Chemiluminescent Substrate (Thermo Fisher Scientific, 34096) and Tanon 5200 Multi‐Automated Chemiluminescent/Fluorescent Image Analysis System. The same experiment was repeated three times independently. Data are presented as mean values ± s.d., n = 3, Two‐tailed Student's t‐tests.

### Testes Grafting

Grafting of cultured and in vivo testes was carried out based on the methods described previously.^[^ ^9^ ^]^ Briefly, Nude mice were anesthetized with 1.2% Avertin (Sigma, T48402) and then castrated. Immediately after castration, 10 dpc testis was grafted under the skin of the left back and 5 d*pp* testis was grafted under the skin of the right back. The same experiment was repeated three times independently. Statistical significance assessed using two‐tailed Student's t‐tests. Data are presented as mean values ± s.d.

### In Vitro Spermatogenesis Culture

The testes of 10 dpc or 5 d*pp* were gently separated into 4‐5 pieces using fine tweezers. Using a standard gas liquid interphase method,^[^ ^11^ ^]^ testicular tissue fragments were placed on 1.5% agarose gels that were half soaked in medium αMEM was supplemented with 10% KSR (Gibco), SCF (20 ng mL^−1^, R&D Systems), bFGF (20 ng mL^−1^, R&D Systems), EGF (20 ng mL^−1^, R&D Systems), GDNF (20 ng mL^−1^, R&D Systems), Activin A (100 ng mL^−1^, R&D Systems), testosterone (10 mm, Acros Organics), FSH (200 ng mL^−1^, Sigma), and BPE (50 mg mL^−1^, Corning Life Sciences). The medium in each well of the agarose gel stand was filled to a level slightly below the upper surface. The medium was refreshed every 2 days, and tissues were cultured at 34°C in 5% CO2. Statistical significance was assessed using Two‐tailed Student's t‐tests. Data are presented as mean values ± s.d.

### Immunofluorescence Staining of Spermatocytes and Chromosome Spreads

Chromosome spreads of prophase I spermatocytes were performed as previously described.^[^ ^70^ ^]^ The primary antibodies utilized in this study were SYCP1 (1:200, Abcam, ab15090) and SYCP3 (1:200, Abcam, ab97672). All testis sections and chromosome spreads stained with these antibodies were subjected to analysis by confocal microscopy (LSM800, Carl Zeiss).

### Round Spermatid Injection (ROSI) and Embryo Transfer

The procedure used for oocyte stimulation has been described elsewhere.^[^ ^71^ ^]^ The round spermatids used for ROSI were obtained from in vitro differentiation of WTSP originally derived from C57BL/6 mice. The oocytes and recipient females used for embryo transfer were of ICR (CD1) background. Briefly, prior to microinjection, MII oocytes were subjected to pre‐stimulation in Ca2+‐free CZB medium (EasyCheck M0000, Nanjing, China) supplemented with 10 mM SrCl2 (Sigma, 439665) for a period of 10 min, followed by exposure to M2 medium containing 5 µg ml^−1^ cytochalasin B (Sigma C6762,) for 5 min. Subsequently, an individual round spermatid cell was microinjected into the pre‐stimulated oocyte using a Piezo‐driven pipette. Following microinjection, the embryos were activated in Ca2+‐free CZB medium containing 10 mm SrCl2 at 37 °C under 5% CO2 for 3–5 h. Finally, the microinjected embryos were transferred into fresh KSOM medium (Sigma–Aldrich MR‐101‐D, Merck, Germany) for in vitro culture. Two‐cell stage embryos were collected and transferred into the oviduct of ICR (CD1) pseudopregnant females, which had been mated with vasectomized males to induce pseudopregnancy. Full‐term pups were delivered naturally.

### CNV

Single cells were isolated using a mouth pipette and placed into lysis buffer. Single‐cell whole‐genome amplification was carried out using the previously described MALBAC method.^[^ ^72^ ^]^ The amplified DNA was subsequently purified using QIAquick PCR Purification Kit (28106, QIAGEN). To prepare libraries, 50 ng of DNA was used along with the TruePrep DNA Library Prep Kit (TD501, Vazyme). The libraries were sequenced on an Illumina NovaSeq 6000 platform in PE150 mode. The CNV regions were identified following the previously established procedures.^[^ ^12^ ^]^

### Cultivation of Human Fetal Gonads

Human fetal gonads were obtained from aborted fetuses with the approval of the Ethical Committee of Nanjing Maternity and Child Health Care Hospital (Permission Number (2017)68). A total of three cultivation experiments were conducted. Two of the specimens were from 24‐week‐old fetuses, and one was from a 25‐week‐old fetus. The gonads from aborted fetuses were carefully dissected into fragments approximately 4–5 mm in diameter using fine tweezers and gently placed on the tips of PEGDA microneedles pre‐soaked in culture medium. The medium volume was carefully adjusted to ensure that the liquid level remained below the hydrogel substrate, preventing complete submersion. Whole testis fragments were cultured at 34.0 °C in a humidified atmosphere of 5% CO₂ in air, with daily medium changes to maintain optimal conditions.

### Statistics and Reproducibility

Statistical tests were performed on GraphPad Prism9.0.0 software. Where appropriate, analysis of variance (one‐way ANOVA) or Two‐sided unpaired t‐test was applied. For experiments with small sample sizes (e.g., n = 3), tests were applied in line with common practice in the field, with appropriate caution in interpretation. In such cases, the Mann–Whitney U test was used as a non‐parametric alternative. All the experiments were performed at least in three biological replicates unless specifically described in the Methods and the figure legends.

### Data and Code Availability

scRNA‐Seq data involved in this study can be accessed on the SRA database, accession numbers PRJNA: 1069296.

## Conflict of Interest

The authors declare no conflict of interest.

## Author Contributions

Y.X., X.Z., B.Z., and M.L. contributed equally to this work. Y.X., X.Z., Y.Y., Y.Z., and J.S. designed experiments. Y.X., X.Z., B.Z., M.L., L.L., L.S. Y.C. D.C. L.L. performed the experiments, data collection and/or data analysis. Y.X., B.Z., M.L., X.G., J.G., Q.C., Y.Y., Y.Z and J.S. wrote the manuscript and incorporated collaborator, manuscript reviewer. Y.Y., Y.Z., and J.S. organized and supervised the entire project.

## Supporting information



Supporting Information

Supporting Information

Supporting Information

Supporting Information

## Data Availability

The data that support the findings of this study are available in the supplementary material of this article.
