# Supplementary material for: A Novel Cultivation System for Germ Cell Proliferation and Sustaining Whole Testicular Niche
Source: Adv Sci (Weinh). 2025 Jul 28;12(39):e02435. doi: 10.1002/advs.202502435 (PMC12533374; doi:10.1002/advs.202502435)
Supplement: Supplementary file 1 — Supporting Information [file ADVS-12-e02435-s001.pdf]

ADVANCED SCIENCE
Open Access

Supporting Information

for *Adv. Sci.*, DOI 10.1002/advs.202502435

A Novel Cultivation System for Germ Cell Proliferation and Sustaining Whole Testicular Niche

Yu Xia, Xiaoxuan Zhang, Bohang Zhang, Minhui Lu, Qing Cheng, Li Liu, Lifang Shi, Yiqiang Cui, Dingdong Chen, Laihua Li, Xuejiang Guo, Jingtao Guo, Jiahao Sha, Yuanjin Zhao* and Yan Yuan**

Figure S1

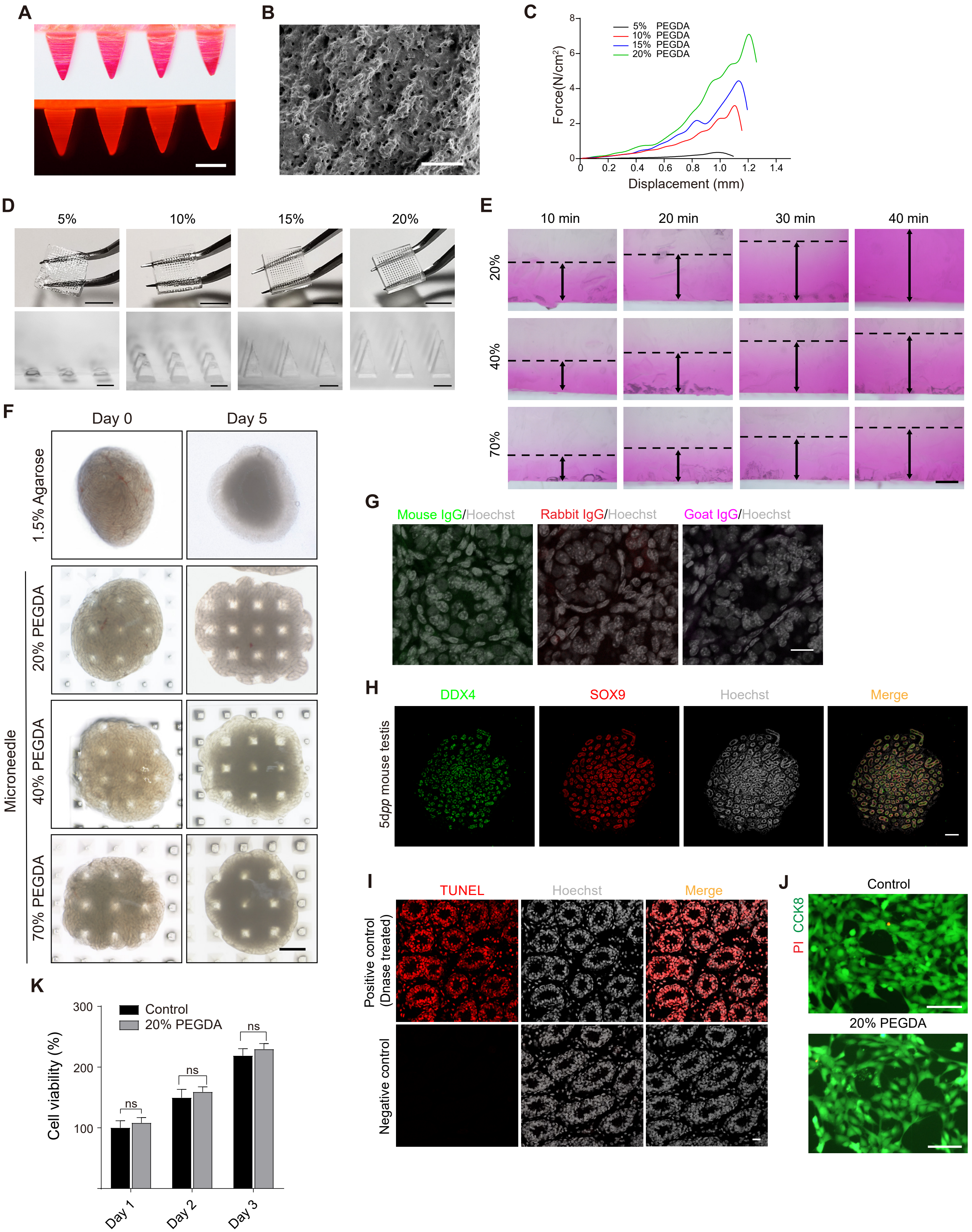

A
B
C
Force(N/cm²)
Displacement (mm)
5% PEGDA
10% PEGDA
15% PEGDA
20% PEGDA
D
5%
10%
15%
20%
E
10 min
20 min
30 min
40 min
20%
40%
70%
F
Day 0
Day 5
1.5% Agarose
Microneedle
20% PEGDA
40% PEGDA
70% PEGDA
G
Mouse IgG/Hoechst
Rabbit IgG/Hoechst
Goat IgG/Hoechst
H
DDX4
SOX9
Hoechst
Merge
5dpp mouse testis
I
TUNEL
Hoechst
Merge
Positive control (Dnase treated)
Negative control
J
Control
20% PEGDA
PI CCK8
K
Cell viability (%)
Control
20% PEGDA
ns
Day 1
Day 2
Day 3

Figure S1. Optical and morphological characterization of PEGDA microneedles, related to Fig. 1.

A, Fluorescence images of PEGDA microneedles with rhodamine B in bright field (up) and dark field (down). Scale bar, 250 μm. **B,** SEM image of the porous surface of PEGDA microneedles. Scale bar, 5 μm. **C,** Graph illustrating the mechanical strength of microneedles at varying PEGDA concentrations. **D,** Optical images of PEGDA microneedles fabricated by different PEGDA concentrations. Scale bars: 5 mm (top), 250 μm (bottom). **E,** Visualization of rhodamine B permeation in agarose mediated by microneedles with various PEGDA concentrations. Scale bar, 1 mm. **F,** Bright-field images of mouse testes cultured using microneedles or agarose. Scale bar, 1 mm. **G,** Immunofluorescence images with isotype IgGs as negative controls. Scale bars, 20 μm. **H,** Immunofluorescence images of 5 d*pp* mouse testis with DDX4 (green), SOX9 (red) and the nuclei was stained using Hoechst 33342 (grey). **I,** TUNEL staining (red) of the 5 d*pp* mouse testicular tissue used as control with Hoechst 33342 stained nuclei (grey). Scale bar, 20 μm. **J,** Fluorescence image comparing NIH-3T3 cells in a control group (left) and cocultured with 20% PEGDA microneedles (right) staining with CCK8 (green) and PI (red). Scale bar, 100 μm. **K,** Cell viability assessment using MTT assay for control group and coculture with 20% PEGDA microneedles.

Figure S2

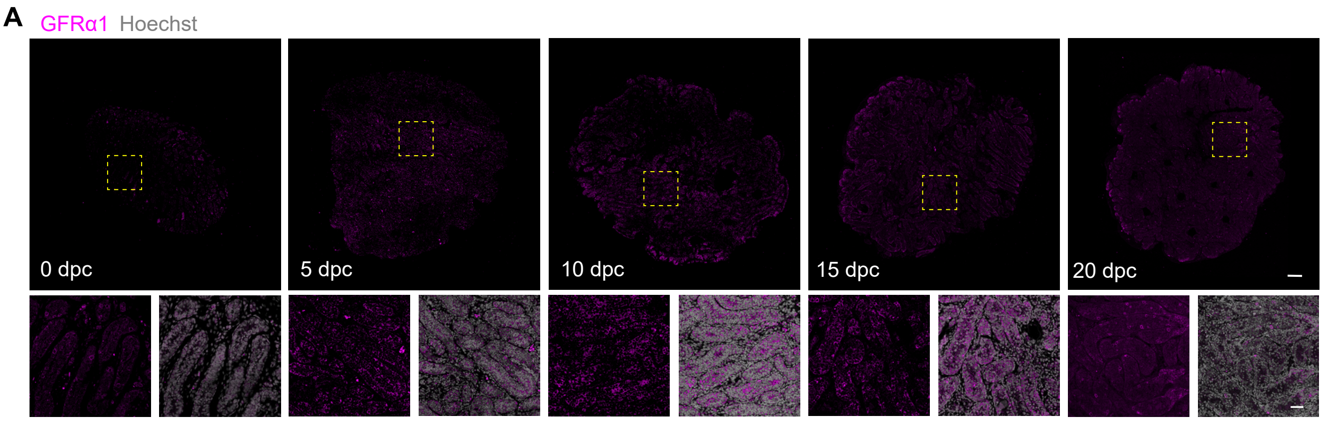

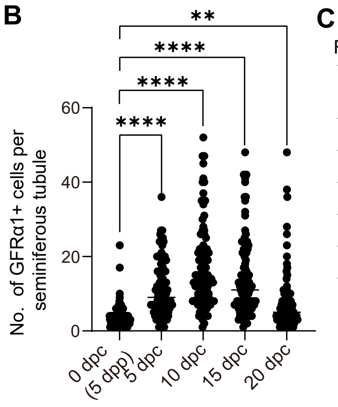

C

Fold increase in average GFRα1+ cells per tubule

Time point	Average GFRα1+ cells	Fold increase (compared to 5 *dpp*)
0 dpc (5 *dpp*)	3.75	-
5 dpc	11.18	2.98
10 dpc	16.48	4.39
15 dpc	13.78	3.67
20 dpc	7.80	2.08

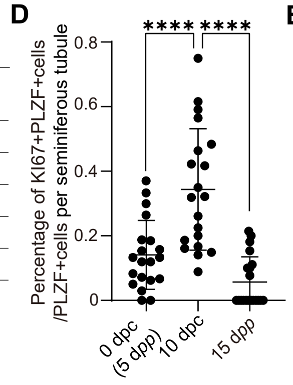

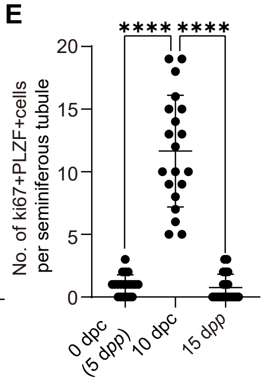

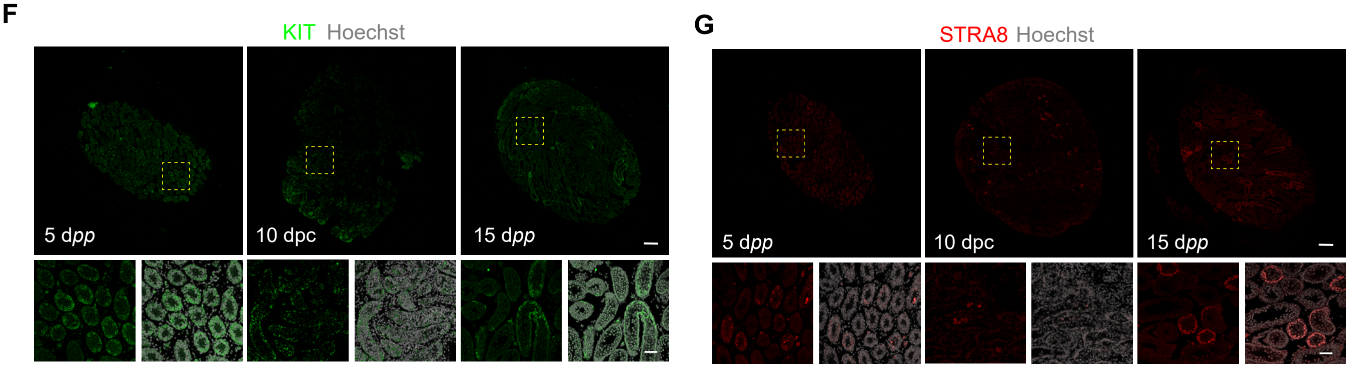

Figure S2. Differentiation status of spermatogonia in WTSP, related to Fig. 2.

A, Immunofluorescence imaging of GFRα1 (magenta) and the nuclei was stained by Hoechst 33342 (grey) in 5 d*pp*, 5 dpc, 10 dpc, 15 dpc and 20 dpc testes. Scale bar, 40 μm. The insets display high-magnification images depicting a representative area of GFRα1 expression in the testes. **B,** Quantification of GFRα1+ cells per seminiferous tubule across different stages. Statistical significance assessed via one-way ANOVA. 5 dpc: $P<0.0001$; 10 dpc: $P<0.0001$; 15 dpc: $P<0.0001$; 20 dpc: $P=0.0027$. Data are presented as mean values ± s.d. **C,** Fold increase in average GFRα1+ cells of 5, 10, 15, 20 dpc testes per tubule compared to 5 d*pp* testis. **D,** Proportion of PLZF+ cells also positive for KI67. 5 d*pp*: $P<0.0001$; 15 d*pp*: $P<0.0001$. **E,** Number of PLZF+ cells also positive for KI67 per seminiferous tubule. 5 d*pp*: $P<0.0001$; 15 d*pp*: $P<0.0001$. Data are presented as mean values ± s.d. **F-G,** Immunofluorescence detection of KIT (green)(E) and STRA8 (red)(F), with the nuclei stained by Hoechst 33342 (grey) in 5 d*pp*, 10 dpc and 15 d*pp* testes. The insets display high-magnification images depicting a representative area of KIT and STRA8 expression in the testes. Scale bars: 200 μm (upper panels), 50 μm (lower panels).

Figure S3

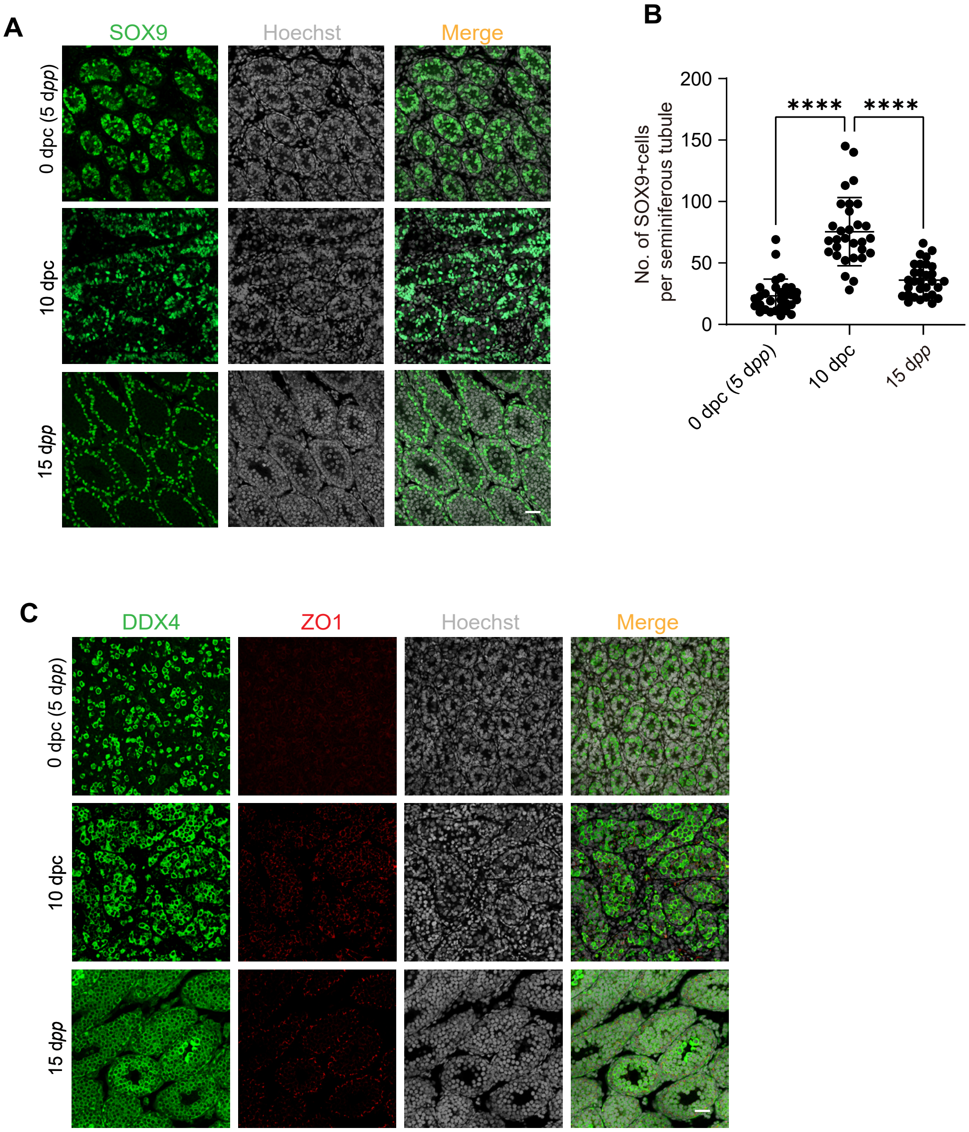
A
SOX9
Hoechst
Merge
0 dpc (5 dpp)
10 dpc
15 dpp
B
No. of SOX9+cells per seminiferous tubule
200
150
100
50
0
****
****
0 dpc (5 dpp)
10 dpc
15 dpp
C
DDX4
ZO1
Hoechst
Merge
0 dpc (5 dpp)
10 dpc
15 dpp

Figure S3. Sertoli cells form the blood-testis barrier in WTSP, related to Fig. 2.

A, Immunofluorescence imaging of SOX9 (green), the nuclei was stained by Hoechst 33342 (grey) in 5 d*pp*, 10 dpc and 15 d*pp* testes. Scale bar, 40 μm. **B,** Quantification of SOX9+ cells per seminiferous tubule in testes at different stages. 5 d*pp*: $P<0.0001$; 15 d*pp*: $P<0.0001$. Data are means ± s.d. **C,** Immunofluorescence imaging of DDX4 (green) and ZO1 (red), the nuclei was stained using Hoechst 33342 (grey) in 5 d*pp*, 10 dpc and 15 d*pp* testes. Scale bar, 40 μm.

Figure S4

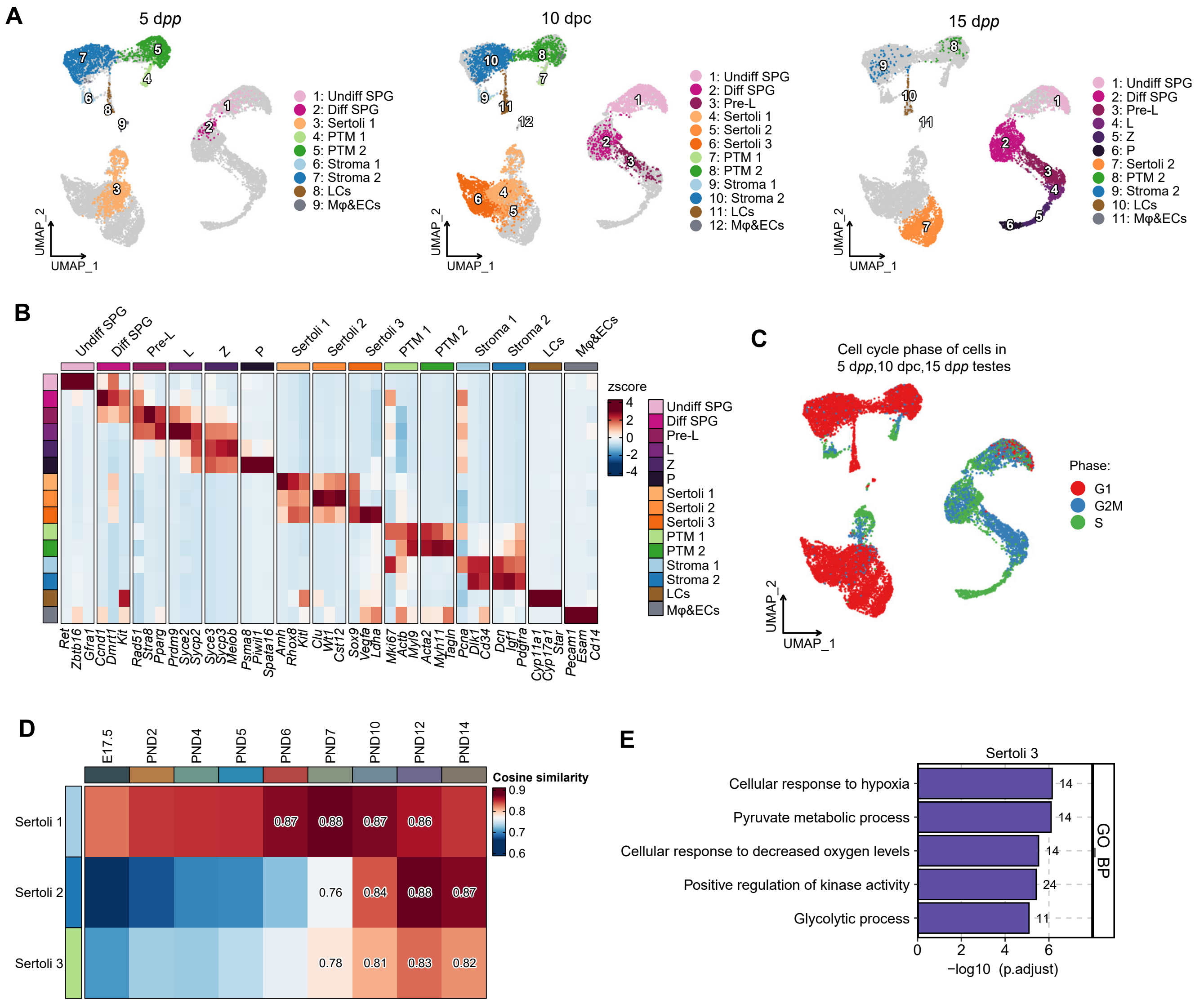

A
5 dpp
1: Undiff SPG
2: Diff SPG
3: Sertoli 1
4: PTM 1
5: PTM 2
6: Stroma 1
7: Stroma 2
8: LCs
9: Mφ&ECs
10 dpc
1: Undiff SPG
2: Diff SPG
3: Pre-L
4: Sertoli 1
5: Sertoli 2
6: Sertoli 3
7: PTM 1
8: PTM 2
9: Stroma 1
10: Stroma 2
11: LCs
12: Mφ&ECs
15 dpp
1: Undiff SPG
2: Diff SPG
3: Pre-L
4: L
5: Z
6: P
7: Sertoli 2
8: PTM 2
9: Stroma 2
10: LCs
11: Mφ&ECs
UMAP_2
UMAP_1
B
Undiff SPG
Diff SPG
Pre-L
L
Z
P
Sertoli 1
Sertoli 2
Sertoli 3
PTM 1
PTM 2
Stroma 1
Stroma 2
LCs
Mφ&ECs
zscore
4
2
0
-2
-4
Ret
Zbtb16
Gfra1
Ccnd1
Dmrt1
Kit
Rad51
Stra8
Pparg
Prdm9
Syce2
Sycp2
Syce3
Sycp3
Meiob
Psma8
Piwil1
Spata16
Amh
Rhox8
Kitl
Clu
Wt1
Cst12
Sox9
Vegfa
Ldha
Mki67
Actb
Myl9
Acta2
Myh11
Tagln
Pcna
Dlk1
Cd34
Dcn
Igf1
Pdgfra
Cyp11a1
Cyp17a1
Star
Pecam1
Esam
Cd14
C
Cell cycle phase of cells in
5 dpp,10 dpc,15 dpp testes
Phase:
G1
G2M
S
UMAP_2
UMAP_1
D
E17.5
PND2
PND4
PND5
PND6
PND7
PND10
PND12
PND14
Cosine similarity
0.9
0.8
0.7
0.6
Sertoli 1
0.87
0.88
0.87
0.86
Sertoli 2
0.76
0.84
0.88
0.87
Sertoli 3
0.78
0.81
0.83
0.82
E
Sertoli 3
Cellular response to hypoxia
14
Pyruvate metabolic process
14
Cellular response to decreased oxygen levels
14
Positive regulation of kinase activity
24
Glycolytic process
11
GO_BP
0
2
4
6
−log10 (p.adjust)

Figure S4. Single-cell transcriptomic profiles of mouse testes from *in vivo* and *in vitro* culture, related to Fig. 3.

A, Partitioned combined UMAP analysis from Figure 3A, with distinct UMAPs for each sample and cell type, color-coded accordingly. **B,** Heatmap showing representative differentially expressed genes for each cell cluster derived from Figure 3A. **C,** Cell cycle phase prediction on UMAP plot from Figure 3A. **D,** Comparison of Sertoli cell developmental time points with *in vivo* reference, with colors indicating similarity degrees. **E,** GO term enrichment analysis for upregulated genes in the Sertoli 3 subgroup.

Figure S5

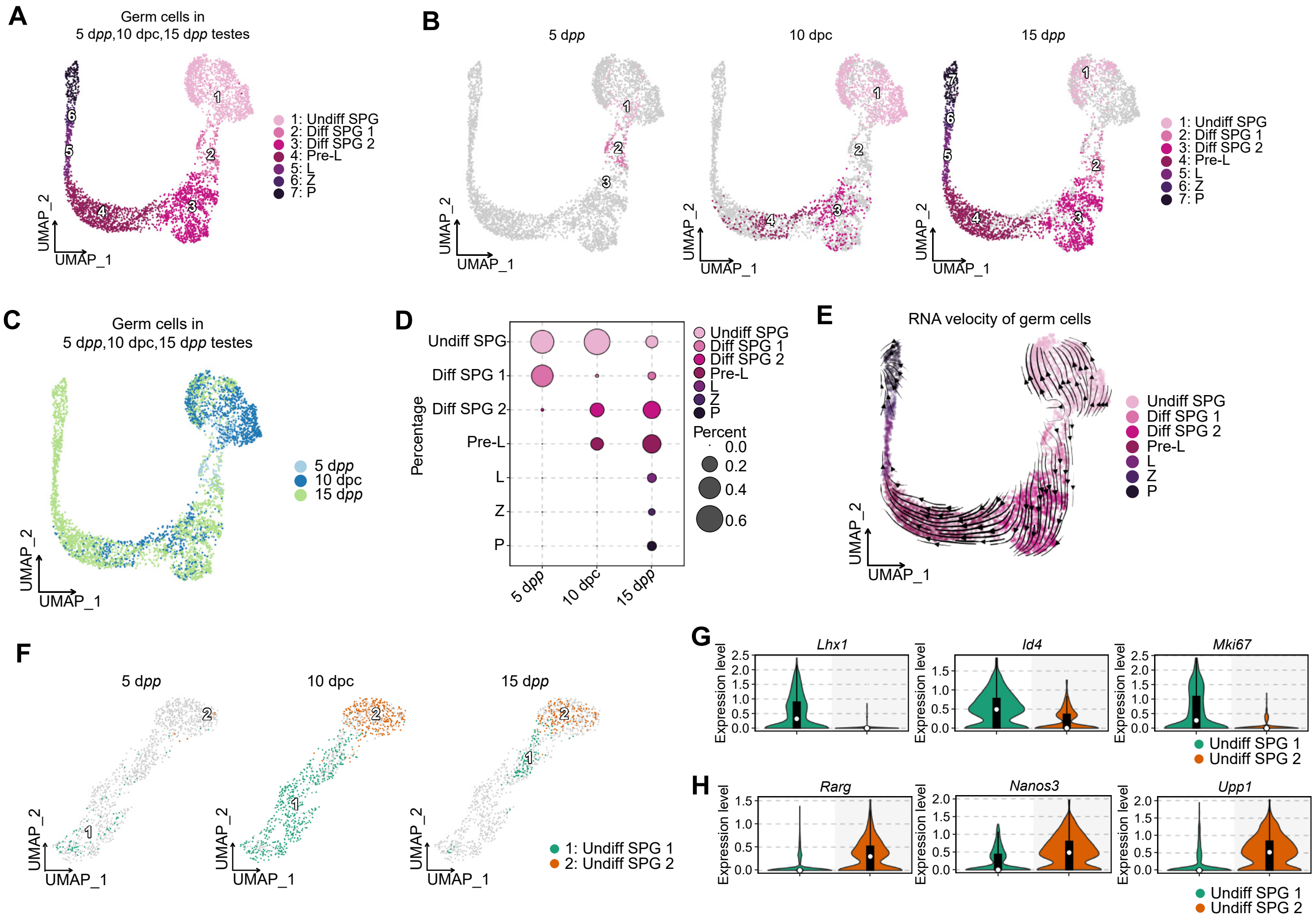
A
Germ cells in
5 dpp,10 dpc,15 dpp testes
1: Undiff SPG
2: Diff SPG 1
3: Diff SPG 2
4: Pre-L
5: L
6: Z
7: P
UMAP_1
UMAP_2
B
5 dpp
10 dpc
15 dpp
C
Germ cells in
5 dpp,10 dpc,15 dpp testes
5 dpp
10 dpc
15 dpp
D
Undiff SPG
Diff SPG 1
Diff SPG 2
Pre-L
L
Z
P
Percentage
Percent
0.0
0.2
0.4
0.6
E
RNA velocity of germ cells
F
1: Undiff SPG 1
2: Undiff SPG 2
G
Lhx1
Id4
Mki67
Expression level
Undiff SPG 1
Undiff SPG 2
H
Rarg
Nanos3
Upp1

Figure S5. Differential single-cell transcriptomic states of germ cells in mouse testes from *in vivo* and *in vitro* culture, related to Fig. 3.

A, Focused analysis (UMAP) of germ cells population, color-coded by cell type identity. **B,** Partitioned combined UMAP analysis of germ cells from different samples, with separate UMAPs for each sample and germ cell type. **C,** UMAP of the germ cells population, color-coded by different sample origins. **D,** Circular representation of cell percentages for each germ cell cluster in different condition samples. **E,** UMAP plot of germ cells annotated with RNA velocity. **F,** Partitioned combined UMAP analysis from Figure 3E, color-coded with distinct UMAPs for each sample and cell type. **G-H,** Violin plot showing expression profiles of marker genes for Undiff SPG 1. and Undiff SPG 2.

Figure S6

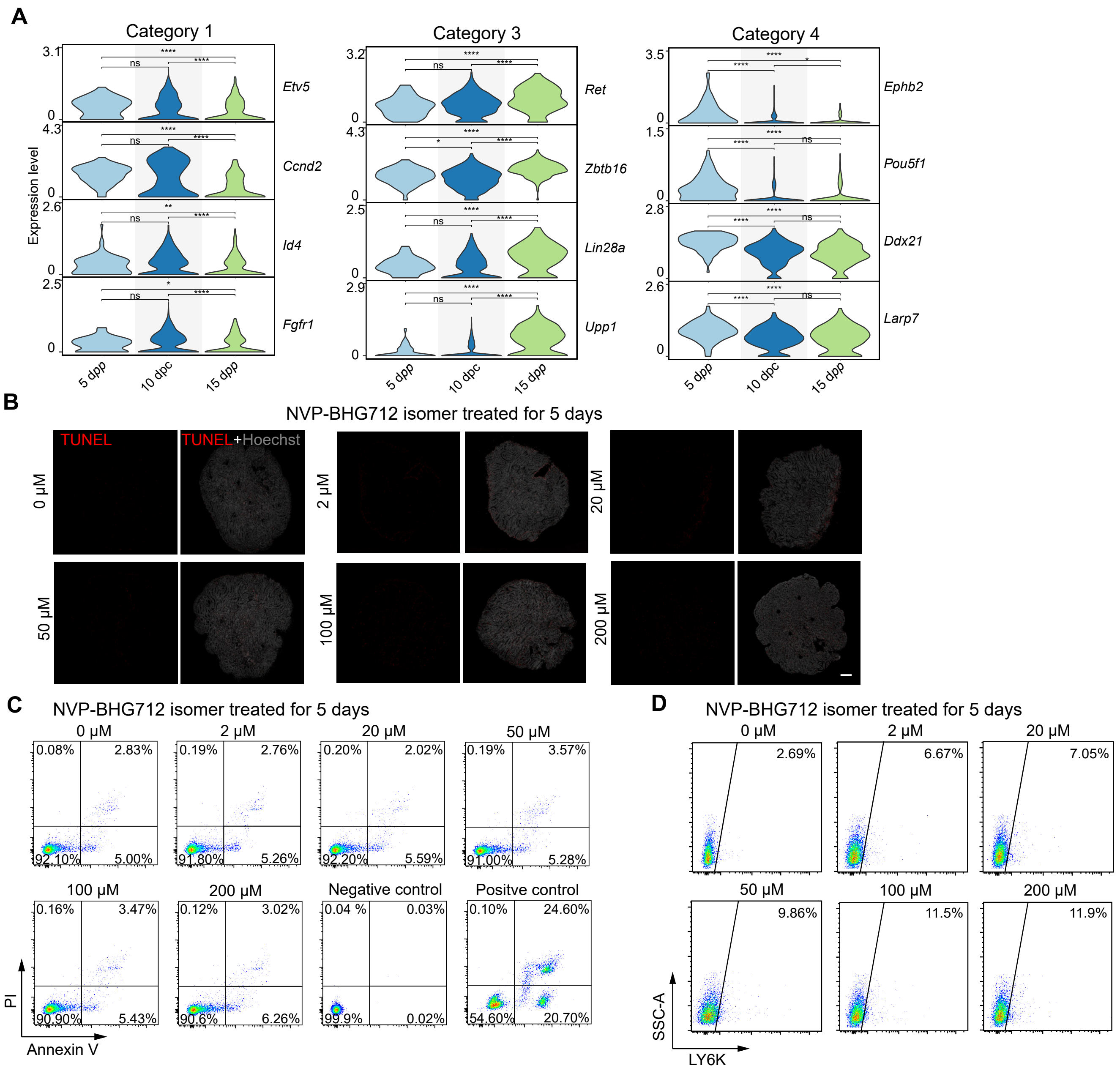

A
Category 1
Category 3
Category 4
Expression level
Etv5
Ccnd2
Id4
Fgfr1
Ret
Zbtb16
Lin28a
Upp1
Ephb2
Pou5f1
Ddx21
Larp7
5 dpp
10 dpc
15 dpp
B
NVP-BHG712 isomer treated for 5 days
TUNEL
TUNEL+Hoechst
0 μM
2 μM
20 μM
50 μM
100 μM
200 μM
C
NVP-BHG712 isomer treated for 5 days
0 μM
0.08%
2.83%
92.10%
5.00%
2 μM
0.19%
2.76%
91.80%
5.26%
20 μM
0.20%
2.02%
92.20%
5.59%
50 μM
0.19%
3.57%
91.00%
5.28%
100 μM
0.16%
3.47%
90.90%
5.43%
200 μM
0.12%
3.02%
90.6%
6.26%
Negative control
0.04 %
0.03%
99.9%
0.02%
Positve control
0.10%
24.60%
54.60%
20.70%
PI
Annexin V
D
NVP-BHG712 isomer treated for 5 days
0 μM
2.69%
2 μM
6.67%
20 μM
7.05%
50 μM
9.86%
100 μM
11.5%
200 μM
11.9%
SSC-A
LY6K

Figure S6. Inhibition of EPHA in WTSP, related to Fig. 4.

A, Violin plot showing expression levels of genes related to Undiff SPG proliferation (Category 1, Category 3 and Category 4) in different samples (5d*pp*, 10dpc and 15d*pp*). **B,** TUNEL staining (red) of 5 dpc testis after NVP BHG712 isomer treatment, with Hoechst 33342 stained nuclei (grey). **C,** FACS-detected percentages of PI, Annexin V positive cells in 5 dpc testes after NVP BHG712 isomer treatment. Hydrogen peroxide-treated testicular cells used as a positive control. **D,** FACS-detected LY6K expression in 5 dpc testis after NVP BHG712 isomer treatment.

Figure S7

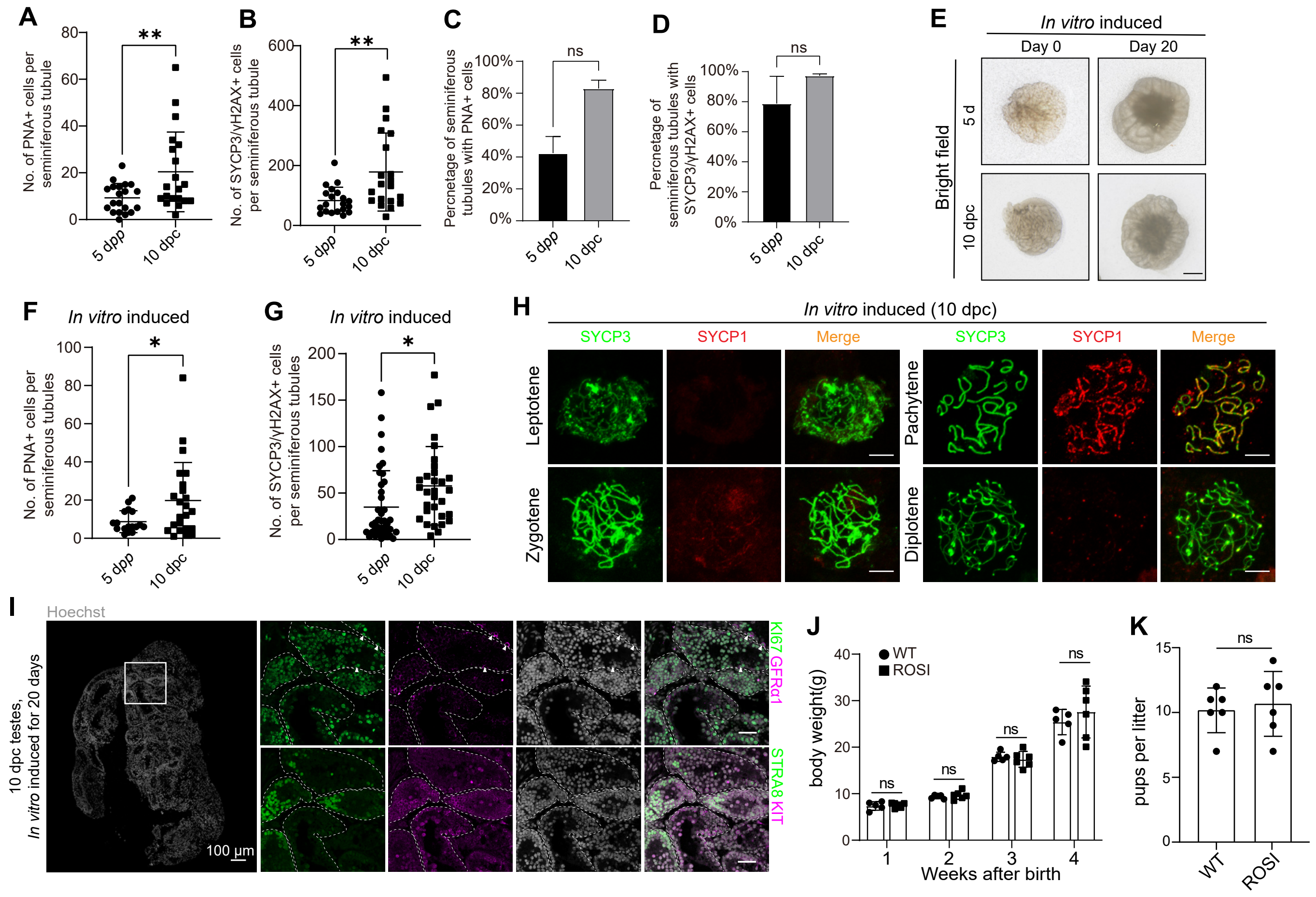
A
No. of PNA+ cells per seminiferous tubule
5 dpp
10 dpc
B
No. of SYCP3/γH2AX+ cells per seminiferous tubule
C
Percnetage of seminiferous tubules with PNA+ cells
D
Percnetage of seminiferous tubules with SYCP3/γH2AX+ cells
E
In vitro induced
Day 0
Day 20
Bright field
5 d
10 dpc
F
In vitro induced
No. of PNA+ cells per seminiferous tubules
G
In vitro induced
No. of SYCP3/γH2AX+ cells per seminiferous tubules
H
In vitro induced (10 dpc)
SYCP3
SYCP1
Merge
Leptotene
Zygotene
Pachytene
Diplotene
I
Hoechst
10 dpc testes, In vitro induced for 20 days
100 μm
KI67 GFRα1
STRA8 KIT
J
WT
ROSI
body weight(g)
Weeks after birth
K
pups per litter
WT
ROSI

Figure S7. ***In vivo* and *in vitro* spermatogenesis of WTSP, related to Fig. 5.**

A-B, Quantification of spermatids (PNA+, P=0.0093) and spermatocytes (γH2AX/SYCP3, P=0.0036) per seminiferous tubule in transplanted testes. Data are presented as mean values ± s.d., Two-tailed Student's t-tests. **C-D,** Quantification of the seminiferous tubules with spermatids (PNA+, P=0.1) and spermatocytes (γH2AX/SYCP3, P=0.1) in transplanted testes. Data are presented as mean values ± s.d., n=3, Mann–Whitney U test. **E,** Bright-field images of testicular fragments of testes cultured with *in vitro* differentiation medium on agarose. Scale bar, 500 µm. **F-G,** Quantification of spermatids (PNA+, P=0.0316) and spermatocytes (γH2AX/SYCP3, P=0.0219) per seminiferous tubule in testes differentiated *in vitro*. Data are presented as mean values ± s.d., Two-tailed Student's t-tests. **H,** Location of SYCP3 (green) and SYCP1 (red) in nuclear spreads of germ cells of 10 dpc-induced testes at 20 days. Scale bar, 5 µm. **I,** Immunofluorescence detection of KI67 (green), GFRα1 (magenta), STRA8 (green), KIT (magenta) from *in vitro* differentiation of 10 dpc testes for 20 days. SSCs in proliferative state are indicated by white arrows. The insets display high-magnification images depicting a representative area (from serial paraffin sections) of KI67/ GFRα1 and STRA8/KIT expression in the testes. The white arrows indicate the typical co-expression of KI67 and GFRA1, signifying self-renewing SSCs. Scale bar, 20 µm. Scale bars: 100 µm (left panels), 40 µm (right panels). **J,** Weight comparison of offspring from *in vitro*-induced spermatids and wild-type mice. Week 1: P=0.7302; Week 2: P=0.4608; Week 3: P=0.5653; Week 4: P=0.9481; Data are presented as mean values ± s.d., Two-tailed Student's t-tests. **K,** Fertility assessment of offspring from *in vitro*-induced spermatids. P=0.6954. Data are presented as mean values ± s.d., Two-tailed Student's t-tests.

Table S1, related to Fig. 2.

Fold increase in average PLZF+ cells per tubule

Time point	Average PLZF+ cells	Fold increase (compared to 5 d*pp*)	Fold increase (compared to *in vivo* counterpart)
0 dpc (5 d*pp*)	11.03	-	-
5 dpc	46.96	4.26 (to 5 d*pp*)	5.06 (to 10 d*pp*)
10 dpc	54.46	4.94 (to 5 d*pp*)	6.49 (to 15 d*pp*)
15 dpc	34.61	3.14 (to 5 d*pp*)	6.41 (to 20 d*pp*)
20 dpc	28.02	2.54 (to 5 d*pp*)	4.48 (to 25 d*pp*)

Table S2, related to Fig. 5.

Development of embryos generated via ROSI *in vitro*-differentiated haploid spermatids

Origin of spermatids	Injected Oocytes(n)	Fertilized oocytes with 2PB and 2PN(n)(%)	Embryos transferred(n)	Live offspring(n)(%)
Spermatids from normal testes cultured	60	36 (60%)	36	6 (16.7%)
Spermatids from 10 dpc testes cultured	48	23 (47.9%)	23	6 (26.1%)
